# Developmental shifts in computations used to detect environmental controllability

**DOI:** 10.1371/journal.pcbi.1010120

**Published:** 2022-06-01

**Authors:** Hillary A. Raab, Careen Foord, Romain Ligneul, Catherine A. Hartley

**Affiliations:** 1 Department of Psychology, New York University, New York, New York, United States of America; 2 Center for Neural Science, New York University, New York, New York, United States of America; 3 Champalimaud Research, Champalimaud Center for the Unknown, Lisbon, Portugal; University College London, UNITED KINGDOM

## Abstract

Accurate assessment of environmental controllability enables individuals to adaptively adjust their behavior—exploiting rewards when desirable outcomes are contingent upon their actions and minimizing costly deliberation when their actions are inconsequential. However, it remains unclear how estimation of environmental controllability changes from childhood to adulthood. Ninety participants (ages 8–25) completed a task that covertly alternated between controllable and uncontrollable conditions, requiring them to explore different actions to discover the current degree of environmental controllability. We found that while children were able to distinguish controllable and uncontrollable conditions, accuracy of controllability assessments improved with age. Computational modeling revealed that whereas younger participants’ controllability assessments relied on evidence gleaned through random exploration, older participants more effectively recruited their task structure knowledge to make highly informative interventions. Age-related improvements in working memory mediated this qualitative shift toward increased use of an inferential strategy. Collectively, these findings reveal an age-related shift in the cognitive processes engaged to assess environmental controllability. Improved detection of environmental controllability may foster increasingly adaptive behavior over development by revealing when actions can be leveraged for one’s benefit.

## Author summary

The ability to determine when one’s actions are consequential organizes learning and decision making across the lifespan. However, few studies have examined how the ability to detect control over our environment changes from childhood to adulthood. Here, we leveraged a computational modeling framework to characterize the component learning processes underlying controllability assessment in children, adolescents, and adults. We observed age-related improvements in controllability assessment that stemmed from an increasing ability to represent contingencies between states and actions and to use that knowledge to make informative interventions that yield diagnostic evidence of the current degree of control. Increasing ability to accurately assess environmental controllability may confer greater recognition of opportunities to adaptively pursue rewards through goal-directed action across development.

## Introduction

Over the course of our lives, we are faced with the challenge of determining when our actions are consequential. In environments that are highly controllable, our actions can reliably produce a particular outcome, whereas in uncontrollable environments, our actions have no causal influence. By estimating the degree of contingency between actions and their resulting outcomes, individuals can assess the extent of control they have over their environment, and adapt their behavior accordingly [1–6]. For example, imagine a child whose parents typically reward her good behavior at the dinner table with dessert. This child might be on her best behavior when eating with her parents because she has learned that her actions directly influence the likelihood of her getting a treat. When at her friend’s house, this same girl might assume that, just like at home, her behavior at the table will determine whether she can have dessert. Thus, she might expend energy minding her manners even if, in actuality, meals at her friend’s home always finish with dessert. Rather than simply generalizing prior beliefs about the controllability of the environment, individuals can make informative interventions to reveal the actual degree of contingency between actions and outcomes [7–10]. Returning to the girl in the example, always being on her best behavior when at her friend’s house cannot test her assumption that her table manners are consequential. To disambiguate whether good behavior, or simply an indulgent parent, is responsible for her getting dessert, a more informative intervention would be for her to occasionally behave poorly. By varying her behavior—that is, by exploring—she can obtain better evidence for assessing whether her actions influence the outcomes she receives when the true causal structure of the environment is unknown.

Experiences of environmental control have a profound effect on learning and behavior, shaping developmental trajectories from an early age [3,11,12]. Infants as young as two months old are sensitive to when outcomes are contingent upon their own actions [13], and early contingent social interaction influences diverse aspects of social and cognitive development, including language learning [14,15] and caregiver attachment [16]. Perceptions of control, whether actual or illusory, are often experienced as subjectively rewarding, a phenomenon proposed to underpin an intrinsic motivation for controllability [17,18]. Motivation to exert control is proposed to be a key driver of development [19,20]. Control facilitates learning and memory [21,22], even from a young age [23,24], and artificial agents equipped with a drive to exert control develop more complex action repertoires [25,26]. During adolescence, greater parental independence provides increased opportunity to make autonomous decisions, which may make accurate recognition of the contexts in which one’s actions are most consequential particularly beneficial [27]. Collectively, these findings suggest that detection of environmental controllability provides foundational knowledge about the structure of the environment that supports the development of an individual’s behavioral repertoire.

An extensive literature has investigated the development of causal inference more broadly [28,29]. Preschoolers, and even infants as early as 8 weeks of age, can perform actions to reveal the causal structure of their environments [30–32]. However, younger children often choose less informative interventions in more complex tasks [30,33,34]. The ability to identify and select informative interventions to reveal the causal structure of the environment continues to develop across childhood and into adolescence [34,35]. While making causal interventions to test hypotheses about action-outcome contingencies is an efficient way to learn, it may require the use of a mental model of the environment, an ability that undergoes continued refinement from childhood into young adulthood [36–38]. Working memory, a cognitive process critical for maintaining and manipulating these mental representations of environmental structure [39], shows similar age-related improvements across adolescence [40,41] may underpin both the ability to make informative interventions and to use the resulting evidence to infer causal structures. Thus, developmental improvements in the cognitive processes that support effective intervention strategies and causal inference suggest that accurate assessment of the degree of environmental controllability may also exhibit marked age-related changes from childhood to adulthood. However, given that the ability to control the environment is typically a premise in causal learning studies, it remains unclear how the ability to test the hypothesis of whether or not one’s own actions actually have causal efficacy might change with age.

As children may not be as adept as adults at making informative causal interventions, they may instead rely on other cognitive processes that can effectively support the detection of environmental controllability from an early age. Learning of statistical regularities present in the environment emerges early in development [42–44]. Younger individuals also tend to exhibit increased stochasticity in action selection [45–49]. In lieu of making highly informative interventions, younger individuals may discern the contingency structure of the environment by applying their robust statistical learning abilities to the observations of actions and outcomes generated through random exploration [50,51]. As children inherently have less experience than adults across many situations, the coupling of greater intrinsic behavioral variability with robust statistical learning ability may be particularly advantageous, as it provides a generalizable strategy for detecting control across diverse environments [45].

An environment can be considered controllable to the extent that actions bring about specific state transitions. Such a causal coupling between the actions of an agent and the states of the environment can be estimated by comparing the predictability of upcoming states (e.g., dessert) when only considering previous states (e.g., dinner) versus when considering both previous states and actions (e.g., dinner at which one showed good manners) [52,53]. Incorporating actions into the predictive process will only improve one’s forecasts in controllable environments, where actions causally influence state transitions. Thus, computational models that compare the accuracy of state predictions based on states alone, versus those based on states *and* actions, can provide a formal account of the controllability estimation process [2,54]. Importantly, predictions about upcoming states can be made in different ways depending on the task and the amount of knowledge one has about its underlying structure [2,5,54]. By comparing how distinct computational models fit participants’ behavior at different stages of development, we can characterize the nature of the learning processes through which participants detect environmental controllability.

Here, we asked whether children, adolescents, and adults differed in their ability to detect changes in environmental controllability and to use informative interventions to reveal these shifts in causal structure. Ninety individuals, aged 8–25, performed a child-friendly adaptation of the ‘Explore-and-Predict’ task—a novel task designed to assess individuals’ ability to estimate the degree of environmental controllability in a dynamic, yet predictable, context and how these beliefs are shaped by the informativeness of interventions [54]. Throughout the task, participants flew with one of two pilots on different colored planes around a set of islands. A key feature of the task was that participants’ choices determined where one of the pilots would fly (i.e., the controllable condition), but critically had no influence on the flight path of the other pilot (i.e., the uncontrollable condition). The two pilot conditions alternated covertly throughout the task, and participants were instructed that they could earn more points if they accurately tracked the current condition. On exploratory trials, participants could make active interventions to infer whether their actions were consequential. On prediction trials, participants were asked to report the likely subsequent state, revealing these controllability beliefs. We used computational modeling to assess both the complexity of participants’ task structure representations and the degree to which their estimation process reflected use of an inferential strategy. We hypothesized that the detection of environmental controllability would improve with age, reflecting the acquisition and use of mental models of environmental structure in controllability estimation across development. Whereas adults’ greater ability to maintain complex task structure representations in working memory may facilitate the use of informative interventions to infer controllability, younger individuals may, instead, rely on random exploration to generate evidence that can reveal the contingency structure of the environment.

## Methods

### Ethics statement

This study was approved by the New York University Committee on Activities Involving Human Subjects (IRB #2016–1194). All participants or a parent, in the case of a minor, provided written consent prior to participation. Participants were compensated $15/hour and were instructed that they would receive a bonus payment based on their performance. In reality, all participants earned a $5 bonus regardless of their performance.

### Participants

As we did not know the size of our hypothesized effect, we targeted a sample size of 90 participants, based on previous developmental studies using computational modeling of choice behavior to characterize age-related cognitive changes [35,55–57]. Ninety-three participants, recruited from the New York City metropolitan area, completed questionnaires and a computer-based learning task. We excluded three participants due to technical errors during the task. Our final sample of 90 participants included thirty children (8–12 years old, mean = 10.46, s.d. = 1.55, female n = 15), thirty adolescents (13–17 years old, mean age = 15.44, s.d. = 1.44, female n = 15), and thirty adults (18–25 years old, mean age = 22.06, s.d. = 2.30, female n = 15). The breakdown of participants’ self-identified race was as follows: 35.56% Caucasian/White, 16.67% African American/Black, 25.56% Asian, and 22.22% Mixed Race. 13.33% reported identifying as Hispanic/Latinx. Participants’ total combined family incomes for the previous twelve months ranged from less than $5,000 to $100,000 or greater. Exclusionary criteria for the study included colorblindness, a diagnosis of psychiatric or learning disorders or disabilities, or the current use of psychoactive medications. All participants had normal or corrected-to-normal vision.

### Assessment of control task

**Fig 1:**
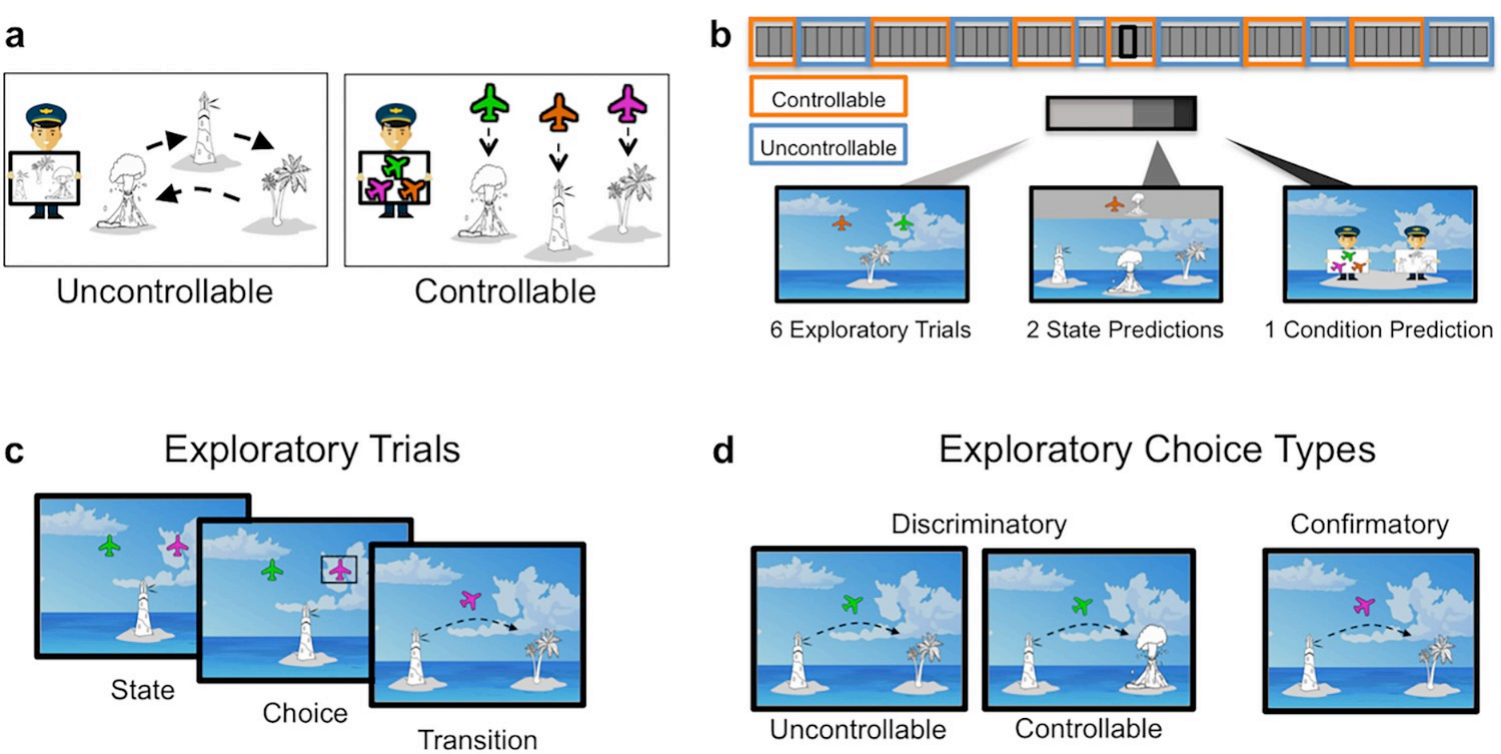
Task Design. (a) Two pilots fly participants around a set of islands. In the controllable condition, the pilot flies to an island based on the color of the plane that the participant selected, allowing participants’ choices to influence the subsequent state. In the uncontrollable condition the pilot flies in a particular route without considering the plane that the participant selected. (b) A schematic of the task is shown. The controllable and uncontrollable conditions of the task alternate covertly. After every six exploratory trials, participants are probed about the subsequent state (“Where is the plane most likely to fly next?”) and the condition (“Which pilot was flying the plane?”). (c) On every exploratory trial, participants see the current island and two planes. They can choose one plane to see where it will fly. (d) Participants can use their knowledge of the transition structure during the exploratory trials to select choices that reveal which pilot is flying the plane. Only discriminatory choices can be used to disentangle the current condition.

We assessed participants’ ability to detect the causal structure of their environment using a task that covertly switched between a controllable and uncontrollable condition. This task was adapted from a previous version of the paradigm created for adults [54]. To make the study suitable for children, we decreased the complexity of the task by removing a second set of controllable and uncontrollable task transitions and included a child-friendly narrative. Participants acted as a travel guide for other passengers by providing them information about flights to three destinations (i.e., the volcano island; the palm tree island; the lighthouse island). There were two pilots, representing the controllable or uncontrollable condition, who could fly passengers around on three different colored planes (i.e., pink, green, orange). One of the pilots flew to the destination based on a specific route, whereas the other pilot flew to the destination depending on the color of the plane chosen (Fig 1A). Participants were never told which pilot was flying the plane, encouraging them to discover the current condition through their choices on exploratory trials. The participant’s goal was to learn where the plane would fly and which pilot was currently flying, in order to help other passengers find their way (Fig 1B). Participants were never told that the two pilots reflected controllability conditions, and the concept of controllability was not introduced in the instructions. To provide a general incentive to perform well throughout the task, participants were told that they would earn treasure for helping other passengers successfully reach their destinations, and that the more treasure they earned, the more bonus money they would receive. To avoid any potential age differences in the subjective value of the bonus, participants were not told the amount of bonus money that they could possibly earn or the conversion rate of treasure into money. The computerized task was coded in Psychtoolbox v3.0.14 with Matlab v2016b.

### Exploratory trials

On each exploratory trial, participants saw the current island and two planes (Fig 1C). Participants chose one of the planes to see where it would fly. Notably, each pilot flew according to a particular rule. In the controllable condition, the color of the plane that the participant selected determined the destination. Thus, participants’ choices determined where the “color” pilot would fly (e.g., selecting the pink plane led to the palm tree, whereas selecting the green plane led to the volcano). In the uncontrollable condition, the pilot flew around the islands according to a particular route. The “route” pilot flew to the next destination in the route irrespective of the color plane that the participant had selected (e.g., volcano to the lighthouse to the palm tree). Prior to playing the game, participants were explicitly instructed about the flight routes that governed each pilot’s flying patterns. As the pilots alternated covertly, participants could use the knowledge about the transition structure for each pilot to discover the current condition. Critically, only one of the two planes displayed on each exploratory trial could be diagnostic as to the current condition. Choosing the other plane was not informative, as it flew to the same island in both the controllable and uncontrollable conditions. In the example shown in Fig 1C, selecting the pink plane would lead to the palm tree in the controllable condition *and* in the uncontrollable condition. Thus, from that departure island, selecting the pink plane contributes to learning transition probabilities but is uninformative as to the current condition. Instead, the green plane is the informative choice (Fig 1D). In the uncontrollable condition, selecting the green plane would lead to the palm tree, because the “route” pilot visits the palm tree after the lighthouse. However, in the controllable condition, the green plane would lead to the island with the volcano. Only by selecting this diagnostic choice (i.e., the green plane) could participants obtain information that enables discrimination between conditions.

The controllable and uncontrollable conditions alternated eleven times during the 360 exploratory trials of the task. Although the total number of exploratory trials remained the same for all participants, the number of exploratory trials prior to each shift in condition was variable (between 18 to 42 trials), and the order of these condition intervals was shuffled between participants (i.e., all participants experience the same numbers of exploratory trials prior to condition shifts, just in a different sequence). Participants were prompted to take a short break three times during the task, resulting in four runs. The length of each run also varied between participants and consisted of no fewer than 72 exploratory trials and no greater than 108 exploratory trials. Following the break, they were reminded of the transition structures for both conditions.

In order to make learning more challenging, the transitions were probabilistic throughout the four runs of the task. Participants were told that sometimes air traffic control instructed the pilot where to fly, but that most of the time pilots followed the usual pattern. During the first and third runs, the planes transitioned as described above 90% of the time, and went to each of the other two islands (the current and other “off-rule” islands) 5% of the time. During the second and fourth runs, we changed the transition probabilities to make the task more difficult. The planes transitioned as described above 80% of the time, and to each of the other two islands 10% of the time. Shifts in these transition probabilities were not instructed. Whereas previous studies have manipulated controllability by changing the probability that an action will yield a specific reward outcome [2,58], this task equates state-transition probabilities across the controllable and uncontrollable conditions, eliminating the potential confound of coupling controllability with predictability [53].

### State predictions

After every six exploratory trials, participants were asked to predict where a particular plane would fly next. Participants were sequentially asked about the two different colored planes that could depart from a given island. In the uncontrollable condition, correct predictions required selecting the same island for both planes because the color of the plane (i.e., the choice the participant made) did not influence which island appeared next. Conversely, accurate responses in the controllable condition required making divergent predictions about where the different colored planes would fly because the next island was contingent upon the color of the plane. In this way, state predictions could reveal the participant’s current beliefs about control. Feedback was randomly given on only one of the two state predictions to incentivize learning without revealing the underlying condition. For reinforced trials, correct predictions yielded an image of a chest full of treasure and incorrect responses led to an image of an empty treasure chest. On the half of trials that were unreinforced, a treasure chest was not displayed. In total, there were 60 prediction pairs (120 state predictions).

### Condition predictions

Following the state prediction pairs, participants were asked which pilot was flying the plane as a direct index of control beliefs that did not depend on accurate knowledge of the task structure. Participants saw a picture of the two pilots side-by-side and then selected the pilot they believed to have been flying the plane, reflecting their beliefs about the current condition. No feedback was given on any of these 60 trials.

### Training phase

Prior to the task, participants were given explicit instructions about where each pilot would fly and had practice playing the game. The training proceeded in the same manner as the task except that all state transitions for the exploratory trials were deterministic and participants were told which pilot was flying the plane. As in the task, participants were asked to predict where the plane would fly and which pilot was flying the plane. In the training, unlike during the task, feedback was given for every prediction. The flight paths for the “color” and “route” pilots were used in both the training and the task. Thus, participants became quite knowledgeable about the structure of the task by the end of the practice. The practice consisted of 48 exploratory trials and eight sets of predictions (where a set of predictions consists of 2 state predictions and 1 condition prediction), split evenly between the controllable and uncontrollable condition.

### Post-task questions

After completing the game, participants answered six questions about the structure of the task. For each pilot, they were asked where they would fly next based on either the color of the plane (controllable condition only) or the current location (uncontrollable condition only). Participants were shown all three destinations and asked to select the correct one for each pilot.

### Secondary measures

As we were interested in the underlying role of working memory in the ability to assess control, participants completed a short list-sorting working memory task from the NIH Toolbox Cognition Battery, which has previously been demonstrated to have high reliability and good construct validity in child and adolescent samples [59]. Participants were shown up to seven items from the same category (e.g., food or animals) displayed one at a time on an iPad. When the image was displayed, participants heard the name of the item. Participants were instructed to repeat the items back to the experimenter in order of increasing size. In the next part of the task, participants were presented with up to 7 items from two distinct categories and asked to sort all the items from one category by size prior to sorting the items from the other category by size. Thus, this task required maintaining and manipulating items in mind in order to perform well. In our analyses, except for the mediation analysis where we used raw scores, we included the age-corrected working memory scores to assess whether differences in working memory, independent of age, were related to behavioral performance. The working memory task was added to the experimental protocol after data collection had begun. Thus, data from one male and five female adolescents are not included in any of the working memory analyses, resulting in a sample size of 84 participants.

To ensure that age was not confounded with differences in age-normed reasoning ability, we also administered the Vocabulary and Matrix Reasoning subtests of the Wechsler Abbreviated Scale of Intelligence (WASI). We observed no significant age differences in age-normed WASI scores [60] (see S1 Appendix).

We also administered the internal locus of control questionnaire to assess subjective sense of control [61] and the MacArthur socioeconomic status questionnaire for exploratory analyses. Results are not reported here.

### Statistical analyses

To examine both linear and quadratic effects of age, we conducted likelihood ratio tests for logistic models and ANOVAs for linear models to determine whether the inclusion of age alone or age and age-squared as predictors in the model provided a significantly better fit. We report which model provided a better fit through model comparison, along with the corresponding statistics from the winning model. Continuous and interval predictors in the regression models were *z*-scored for interpretability. Age was included as a continuous variable, unless otherwise noted, and categorical age bins were applied for visualization. Age-squared was calculated by squaring the *z*-scored age. Behavioral analyses were performed using R version 3.5.2 [62] and Matlab 2016a (Mathworks). All *p*-values reflect a two-tailed alpha threshold of *p* < .05. Mixed-effect models were conducted in R using the *afex* package Version .22–1 [63]. We used the optimizer “bobyqa” and set the number of model iterations to one million. The maximal model was specified to minimize Type I error [64], except where noted. If the maximal model did not converge or resulted in a singular fit, we reduced the random effects structure until the model converged. Details on model specification and full results can be found in S1 Appendix. Mediation analyses were performed using the *mediation* package in R [65]. Confidence intervals were estimated using 10,000 bootstrapped samples to test the significance of the mediation effects.

### Computational modeling

To gain insight into the cognitive mechanisms underlying participants’ choices and the source of potential age-related differences, we fit four models (the Spectator, Actor, Learned Transition Structure, and Task Set models) inspired by a previous study in adults using a more complex version of the task [54]. These models formalize different ways of making predictions about the state that will be encountered next (*s*′)—by estimating transition probabilities based on states (Spectator model) or states and actions (Actor model); by estimating controllability based on the difference between the Spectator and Actor predictions (Learned Transition Structure model); or by inferring controllability based on perfect knowledge of the task transition structure (Task Set model), which affords the ability to make diagnostic interventions from the start of the task. The Task Set model is the only model that formalizes an inferential strategy and requires representation of the complete task structure in working memory. Thus, the set of models requires tracking an increasingly large set of transition probabilities, with inference requiring the maintenance and manipulation of these complex structures in working memory.

For all but the Task Set model, predictions for transitions that were experienced are updated using an error-driven process. The prediction error captures the difference between the experienced transition (which was coded as 1) and the predicted transition probability. The extent to which learned transition probabilities are updated by the most recent prediction error is governed by the learning rate (*α*). Predictions for the transitions that did not occur are decremented, ensuring that transition probabilities sum to 1 (see S2 Appendix for details).

The Spectator model makes predictions about the subsequent state (*P*(*s*′|*s*)) based solely on states (*s*), without taking actions into account, thus assuming environmental uncontrollability; Eq 1).


$$P(s\prime |s)\leftarrow P(s\prime |s)+\alpha_{ss\prime }(1-P(s\prime |s))$$ (1)

The Actor model makes these predictions (*P*(*s*′|*s*, *a*)) based on states (*s*) and actions (*a*), thus assuming environmental controllability; Eq 2).


$$P(s\prime |s,a)\leftarrow P(s\prime |s,a)+\alpha_{sas\prime }(1-P(s\prime |s,a))$$ (2)

The remaining two models, the Learned Transition Structure model and the Task Set model, both dynamically estimate the causal influence of actions over state transitions by comparing predictions about subsequent transitions from the Spectator and Actor models (*P*(*s*′|*s*, *a*)−*P*(*s*′|*s*)). This expected difference, Ω, represents an online estimate of the degree of controllability of the environment. In a controllable environment, actions contribute to predictions about the upcoming state and therefore, there will be an action for which *P*(*s*′|*s*, *a*) > *P*(*s*′|*s*). Higher *Ω* values provide evidence that the environment is more controllable. Unlike the Spectator and Actor models, both of the controllability models have a second-order learning rate (*α*_*Ω*_), which governs the updating of the expected difference in the predictive capability of the Spectator versus the Actor model (i.e. *P*(*s*′|*s*, *a*)−*P*(*s*′|*s*); Eq 3).


$$\Omega \leftarrow \Omega +\alpha_{\Omega }(P(s\prime |s,a)-P(s\prime |s)-\Omega )$$ (3)

In order to transform *Ω* into a probability between 0 and 1 that can be used for prediction, and to capture distinct ways in which estimates of control may be biased, Ω is transformed into an “arbitrator”, ⍵, using a two-parameter sigmoidal function (Eq 4).


$$\omega =\frac{1}{1+exp(-\beta_{\Omega }(\Omega -{bias}_{\Omega }))}$$ (4)

The ‘bias’ parameter (bias_Ω_) in the sigmoidal function acts as a threshold above which Ω is interpreted as evidence in favor of a controllable environment, and thus can capture persistent biases toward estimates of controllability or uncontrollability. The slope parameter of the sigmoidal transformation (β_Ω_) determines the extent to which the most likely first-order model (i.e. the Spectator or the Actor depending on whether Ω is above or below the ‘bias’ estimate) is given priority when making predictions about a future state.

For the Learned Transition Structure and Task Set models, state predictions are made by weighting the spectator and actor model using the arbitrator ⍵ (Eq 5).


$$p(S\prime =i)=\omega \;\underset{j=1:3}{\max}p(S\prime =i|S_{j},A)+(1-\omega )p(S\prime =i|S)$$ (5)

The difference between the Learned Transition Structure model and the Task Set model is the manner through which the transition structure is learned. The Learned Transition Structure model updates the state-state and state-action-state transition probabilities from experience, whereas the Task Set model uses prior knowledge about the rules governing the task transition structure to infer the degree of controllability of the environment and make state predictions. Therefore, in the Task Set model, transition probabilities are fully pre-learned and set to either 1 or 0 based on the rules governing state-state and state-action-state transitions that were explicitly instructed during the training phase. As transition probabilities for this model are not updated, the first-order learning rate is set to 0. Within the Task Set model, the Spectator and Actor models explicitly represent the two possible task sets that can alternate covertly during the experiment. The arbitrator, ⍵, derived from Ω, represents the arbitration between task sets when making a prediction. Thus, the Task Set model reflects the use of an inferential (or hypothesis-testing) strategy, as opposed to the Learned Transition Structure model that reflects a continuous updating based on experience.

For all four models, the probability that the participant predicts the next state is determined by a softmax equation (Eq 6). An inverse temperature parameter (β_choice_) controls choice consistency with respect to predicted transitions. Higher values for β_choice_ implies that the participant systematically selected the most likely transition, whereas a value close to 0 implies that the participant randomly guessed on prediction trials.


$$p(prediction=i)=\frac{exp(\beta_{choice}p(S^{\prime }=i))}{\sum_{j=1}^{j=3}exp(\beta_{choice}p(S\prime =i))}$$ (6)

In total, the Learned Transition Structure model has five free parameters: two learning rates, two parameters that transform Ω into ⍵, and an inverse temperature. The Task Set model has only four free parameters, as the first-order learning rate is set to 0. The Spectator model and the Actor model both have two free parameters: a learning rate and an inverse temperature.

Model variables were updated in the same manner following every exploratory trial and on state prediction trials that ended with feedback. However, only state predictions were used to constrain model fits, as there were no correct choices during exploration (even though there were informative or uninformative choices). Bayesian Information Criterion (BIC) was used for model comparison. A full description of the model space, fitting, and model comparison procedures is available in the S2 Appendix (see also S2 Fig).

### Information-theoretic analyses

To characterize age-related differences in the pattern of exploratory choices, we quantified how evenly the space of possible state-action pairs was explored throughout the task. To do so, mutual information was calculated *a posteriori* for each participant by computing the shared information between states and actions for all exploratory trials I(S_t_, A_t_). Greater mutual information reflects greater diversity in action selection, whereas less mutual information reflects a tendency to repeatedly select the same action in a given state. We used the MIToolbox (https://github.com/Craigacp/MIToolbox) to compute mutual information.

## Results

### State prediction accuracy

During the task, participants were probed about where a plane would fly next as an indirect way to assess their beliefs of environmental controllability. We first examined how the accuracy of these revealed assessments of control changed as a function of the true degree of environmental control over the course of the task, as well as how accuracy was modulated by individual differences in working memory, a hypothesized component process of controllability inferences. We fit a generalized mixed-effects logistic regression model to participants’ choices on state prediction trials, collapsing across runs with 80% versus 90% transition probabilities between states (as there were no significant differences; see S1 Appendix). We calculated the proportion of diagnostic choices on every set of six exploratory trials for each participant, reflecting the informativeness of interventions prior to assessing control. Age, condition, state prediction trial number, proportion of diagnostic exploratory choices, and age-corrected working memory were included as predictors in the model. Interactions were included in the model except between working memory and proportion of diagnostic exploratory choices, as we wanted to limit the size of the model and had no *a priori* hypotheses about these interactions. The addition of the quadratic age term did not provide a better fit than the model including linear age alone (*X*^*2*^(12) = 9.77, *p* = .636).

**Fig 2:**
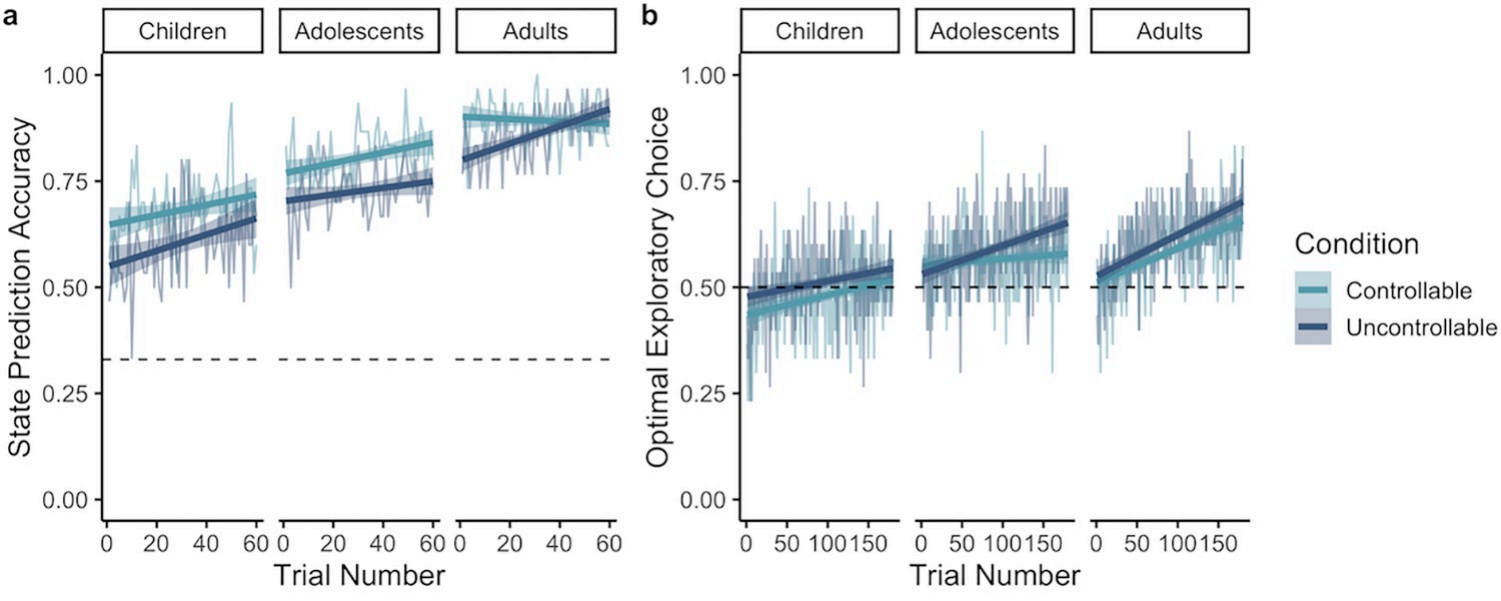
Performance on state predictions and exploratory trials over the course of the task. (a) Accuracy on state predictions and (b) diagnostic choices on exploratory trials are shown as a function of age group (children: 8–12 years, adolescents: 13–17 years, adults: 18–25 years), trial, and condition. Thinner lines represent the average performance for each group and condition, whereas the thicker lines represent the line of best fit with a 95% confidence interval. Chance performance is denoted by dashed lines.

Participants across age were more accurate at making predictions in the controllable condition (*X*^*2*^(1) = 12.71, *p* = .0004; Fig 2A), although performance for both conditions was well above chance even in the youngest individuals. As we hypothesized, state prediction accuracy increased with age (*X*^*2*^(1) = 32.88, *p* < .0001), suggesting that accuracy in assessments of controllability improved across development. Participants’ accuracy improved over the course of the task (*X*^*2*^(1) = 11.35, *p* = .0008), providing evidence of learning. There was also a significant effect of diagnostic choice on state prediction accuracy (*X*^*2*^(1) = 7.3, *p* = .007), such that a greater number of diagnostic interventions led to more accurate controllability assessments. With age, individuals became better at using diagnostic interventions to assess environmental controllability (age-by-diagnostic choice interaction: *X*^*2*^(1) = 7.82, *p* = .005). In addition, diagnostic exploratory choices influenced prediction accuracy differently for the controllable and uncontrollable condition (condition-by-diagnostic choice interaction: *X*^*2*^(1) = 5.07, *p* = .02), with the degree of diagnostic choices promoting accurate state predictions to a greater extent in the uncontrollable condition. This result provides further evidence of participants’ bias toward controllability beliefs, as it suggests that stronger evidence (provided by diagnostic exploratory choices) was needed to confirm that actions were ineffective than to confirm their causal efficacy. No other main effects or interactions reached significance (*p*’s > .07; see Table A in S1 Appendix for a full table of results). We repeated this analysis in the full sample after removing working memory as a predictor, and the same pattern of significant and non-significant effects remained (see Table B in S1 Appendix).

To characterize the effect of reversals of controllability conditions, we examined whether accuracy and response times changed after these shifts in conditions. We performed a mixed-effects logistic regression, including trials since reversal, age, and condition as predictors of state prediction accuracy in the model. We found a significant effect of trials since reversal on state predictions (*X*^*2*^(1) = 4.02, *p* = .04), suggesting that performance is lowest following the reversal and improves with time (see S1 Appendix for model details and full results). No interactions reached significance (*p*’s > .2), suggesting that the temporal dynamics of performance following reversals did not differ across age. Next, we investigated whether response times differed by age, condition or as a function of prediction trials since reversal. We found that participants across age were faster to predict the next state in the controllable condition (see S1 Appendix for analysis details and results). As response times are typically slower when choice uncertainty is higher [66,67], these findings parallel the more accurate performance on state predictions when actions are causal.

### Condition prediction accuracy

In addition to state predictions, which required learning the structure of the task, participants were also asked to make binary judgments about which pilot was flying the plane. These condition predictions acted as a coarser measure of control beliefs, as they did not directly rely on knowledge of the task transition structure. We repeated the mixed-effects logistic regression analysis to predict participants’ choices on condition prediction trials, again collapsing across runs with 80% versus 90% transition probabilities between states (as there were no significant differences; see S1 Appendix). Age, condition, condition prediction trial number, proportion of diagnostic exploratory choices, and age-corrected working memory were included as predictors in the model. As for the state prediction mixed-effects model, we did not allow working memory and proportion of diagnostic exploratory choices to interact. Including an age-squared term in the model did not improve the fit (*X*^*2*^(12) = 4.26, *p* = .978). As with state predictions and in line with our hypothesis, explicit judgments about environmental controllability were more accurate in older individuals (*X*^*2*^(1) = 26.95, *p* < .0001). Accuracy was higher in the controllable condition, which reflected a bias toward beliefs of control, and performance improved over the course of the task (condition: *X*^*2*^(1) = 9.38, *p* = .002; trial: *X*^*2*^(1) = 4.86, *p* = .03). Selecting informative choices during the exploratory trials was associated with more accurate explicit judgments (*X*^*2*^(1) = 17.32, *p* < .0001). As with state predictions, older individuals were significantly better at using diagnostic choices on the exploratory trials to predict the condition (age-by-diagnostic choice interaction: *X*^*2*^(1) = 16.78, *p* < .0001). Greater working memory performance was indicative of more accurate predictions about the condition (*X*^*2*^(1) = 3.73, *p* = .05). None of the other main effects or interactions reached significance (*p*’s > 0.1; see Table C in S1 Appendix for full results). As with state predictions, we repeated this analysis without working memory to allow for a full sample size. All significant effects remained significant and nothing that was previously non-significant became significant (see Table D in S1 Appendix).

To confirm whether our direct measure of controllability (condition predictions) related to our indirect measure of controllability (state predictions), we performed a linear regression with average accuracy on state predictions as a predictor of average accuracy on condition predictions. We found a significant correspondence between the two controllability measures that strengthened slightly with age (state prediction accuracy: *β* = .958, s.e. = .071, *t*(86) = 13.41, *p* < .0001; age-by-state prediction accuracy *β* = .142, s.e. = .071, *t*(86) = 1.996, *p* = .049), reflecting metacognitive awareness about the degree of environmental controllability from middle childhood, which strengthened with age. Unlike for state predictions, we did not find a significant effect of trials since reversal on condition prediction accuracy (*p* > .5). In addition, across age participants were marginally slower to predict the condition in the controllable context (*X*^*2*^(1) = 3.74, *p* = .05; see S1 Appendix for model details and full results for these analyses).

To better assess the specific role of working memory in controllability assessments, we asked whether age-related differences in working memory might account for developmental improvements in condition prediction accuracy. First, we confirmed that raw scores from the working memory task increased with age (*β* = .33, SE = .1, *t* = 3.11 *p* = .003) and related to greater accuracy on condition predictions while controlling for age (*β* = .23, SE = .1, *t* = 2.17 *p* = .033). Then we tested whether working memory mediated this relation between age and condition prediction accuracy. This analysis revealed that working memory partially mediated the relationship between age and condition prediction accuracy (standardized indirect effect: .07, 95% confidence interval: [.005: .19], *p* = .028; standardized direct effect: .34, 95% confidence interval: [.12: .53], *p* = .002), highlighting working memory as a core cognitive mechanism supporting the detection of control. A similar pattern of results was observed for state predictions (see S1 Appendix).

### Exploratory choices

The age differences evident in participants’ inferences of control suggest that individuals might be relying on different intervention strategies on exploratory trials. On every exploratory trial there were two choices, only one of which was diagnostic as to the condition. To investigate the informativeness of interventions during exploratory trials across age, we fit a generalized mixed-effects logistic regression with age, condition, trial, and all interaction terms as predictors. The model that included the addition of an age-squared term provided a better fit (*X*^*2*^(4) = 10.26, *p* = 0.036). Participants selected the diagnostic exploratory choice more in the uncontrollable than controllable condition (*X*^*2*^(1) = 16.05, *p* < .0001; Fig 2B). While we had no *a priori* predictions about such a difference, this effect may be consistent with the bias toward controllability beliefs revealed by participants’ state predictions, and suggest that more evidence was required for participants to confirm that their actions were not causal. A significant effect of trial reflects that knowledge about the task structure may improve over the course of the task, facilitating more frequent diagnostic interventions in the later trials (*X*^*2*^(1) = 7.97, *p* = .005). A significant age-by-trial interaction revealed that adults selected the diagnostic choice more frequently as the task progressed, whereas younger individuals showed less change in selecting the diagnostic choice over the course of the task (*X*^*2*^(1) = 5.24, *p* = .02). No other effects reached significance (see Table E in S1 Appendix for full results). Thus, adults may be better at learning to use knowledge of the task rules to make informative interventions.

The age-related improvement in learning to make diagnostic exploratory choices was paralleled by an age-related increase in the tendency to perform specific actions in specific states of the environment (e.g., repeatedly selecting the green plane when on the palm island). Mutual information, which captures the amount of information gained about actions by knowing about the states in which they were selected (and vice versa), increased with age (rho = 0.42, *p* < 0.001), suggesting that younger individuals tended to evenly explore available actions within a state, while older individuals demonstrated more rigid coupling of states and actions. While adults may rely on an accurate mental representation of the task structure to make informative interventions that are diagnostic as to the current degree of controllability, younger individuals may thus aggregate over state-action-state observations to statistically estimate the degree of causality between their actions and outcomes. By exhibiting greater variability in their exploration of actions, younger individuals can generate a broader set of observations that may facilitate learning from experience, rather than through the use of the explicit task knowledge.

### Computational modeling of behavior

Computational modeling allows us to formally articulate components of the cognitive processes that may contribute to an individual’s choices. Here, we investigated how the computations used to assess environmental controllability changed from childhood to adulthood. We fit four computational models, described in detail in S2 Appendix, to each participant’s data. These models differed in how they evaluated contingencies between states and actions and how they incorporated knowledge about the task structure that could be gleaned from the instructions and the training phase. The Spectator model and the Actor model assumed static beliefs of uncontrollability or controllability, respectively. The Learned Transition Structure model and Task Set model both incorporated dynamic beliefs about environmental controllability, but predictions about the transition structure were updated from experience in the Learned Transition Structure model and from explicit task instructions in the Task Set model.

**Fig 3:**
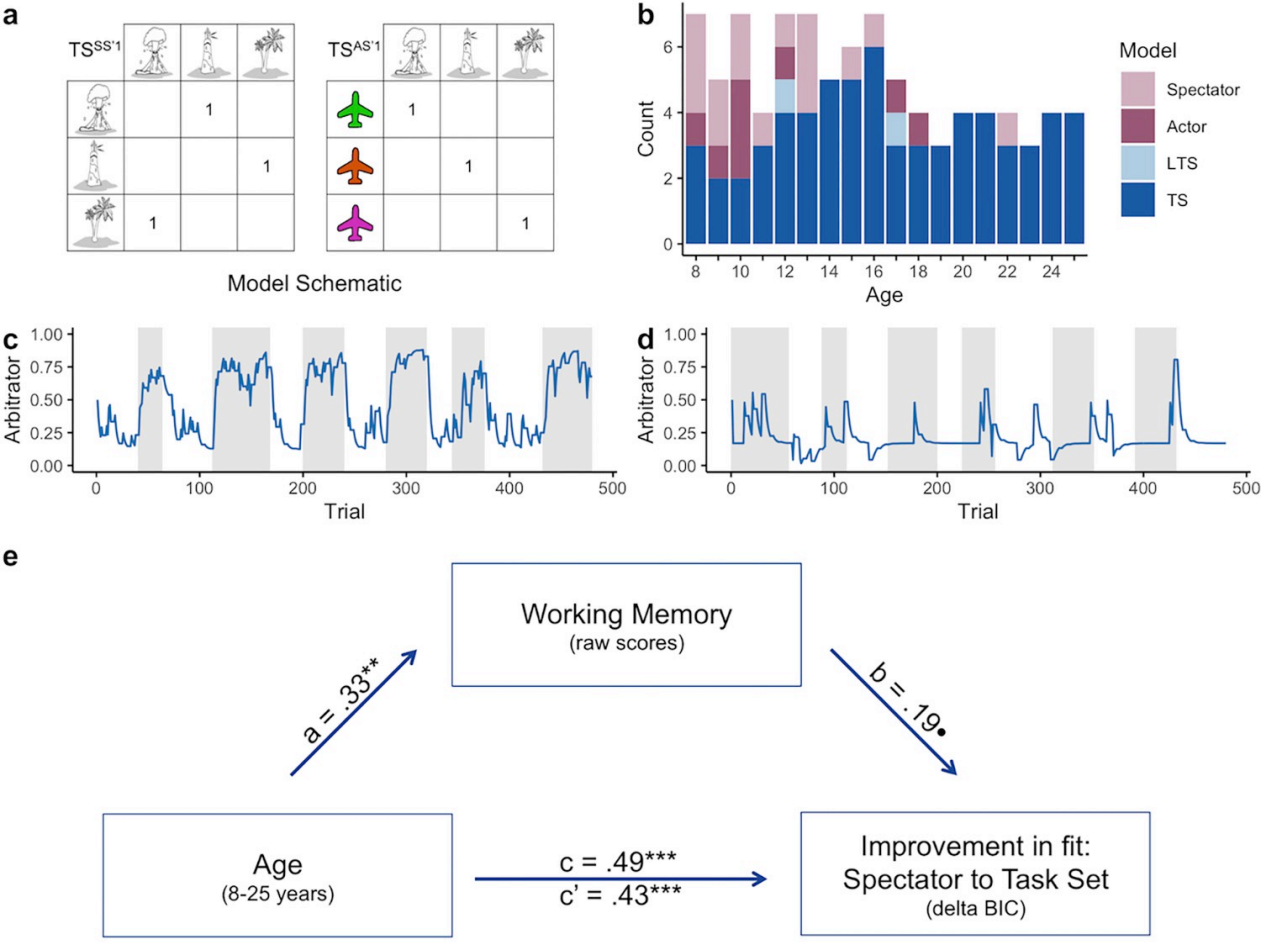
Model comparison and working memory mediation analysis for the best-fitting Task Set Model. (a) Task Set rules that govern how the state-state (SS’1) or state-action-state (SAS’1) models are updated after each trial. (b) The number of participants best fit by each model as a function of age. (c-d) Dynamics of the arbitrator are plotted for two example participants. Higher values reflect greater inferred controllability. Shaded areas reflect the controllable condition and white reflects the uncontrollable condition. (e) Working memory partially mediated the relation between age and improvement in quality of fit between the Spectator and Task Set model. Path a shows the regression coefficient for age-related improvements in working memory. Path b shows the regression coefficient for the influence of working memory on the improvement in model fit between the Spectator and Task Set models, while controlling for age. Path c’ and c show regression coefficients for the influence of age on improvement in fit (Δ BIC) with and without including working memory, respectively. The following abbreviations are used: LTS: Learned Transition Structure model; TS: Task Set model. **Denotes p < .01; ***denotes p < .001; ●p = .068.

At the group level, Bayesian model comparison revealed a clear advantage for the Task Set model as the most frequent model in the population compared to the other models, indicating that the majority of participants made controllability inferences using a hypothesis-testing approach in which the task rules (Fig 3A) are represented explicitly (Fig 3B; exceedance probability > 0.999; estimated frequency: 80.5%; BIC difference with the second best model = 2516). Model and parameter recoverability were high, ensuring interpretability of the computational modeling results (see S2 Appendix and S2 Fig). For analyses on how parameter estimates from the best-fitting model change across age, see Table A in S2 Appendix and S3 Fig. While the majority of children, adolescents, and adults were best fit by the Task Set model, children displayed the greatest strategy heterogeneity. Younger participants had a higher propensity than older individuals to rely on the Spectator model, which did not take into account the effects of one’s actions in the prediction of upcoming states. Importantly, at the group level, all four models fit participants’ choice behavior better than a random model (no learning, 0 free parameters): the overall BIC of the random model greatly exceeded the overall BIC of the worst-fitting model (i.e., the Spectator model; *Δ* BIC: 3707). Examination of the relative advantage of the models within the comparison set, captured by the difference in their BIC values, provides evidence of systematic age-related changes in the information participants took into account when making controllability assessments. A positive correlation between age and the relative advantage of the Actor model over the Spectator model reflects that with age, individuals increasingly incorporated the contingencies between states and actions into their model of the task structure (rho = 0.39, *p* < 0.001; S1A Fig). Furthermore, a significant positive correlation between age and the relative advantage of the Task Set model over the Learned Transition Structure model suggests an increased reliance on explicit rule-based knowledge with age (rho = 0.48, *p* < 0.001; S1B Fig). Thus, individuals both considered the causal influence of their actions, and incorporated knowledge of the rule structure into their mental representation of the task to a greater extent with increasing age.

We next examined model-derived controllability estimates from the best-fitting Task Set model to validate their relation to participants’ controllability assessments. In the model, participants’ inferred controllability beliefs were reflected in the value of the arbitrator (⍵). Whereas estimates of controllability for some participants reflected shifts in inferences of controllability that mirrored the true causal structure of the environment (Fig 3C), others reflected a bias toward uncontrollability beliefs (Fig 3D). We performed a mixed-effects linear regression to investigate how model-derived controllability estimates, measured through the trial-by-trial arbitrator term, changed with age, across conditions, and over the course of the task. Model fit did not improve after including an age-squared term (*X*^*2*^(4) = 5.76, *p* = .218). There was a main effect of condition (*X*^*2*^(1) = 131.49, *p* < .001), with arbitrator values larger in the controllable condition. The difference in the arbitrator values between conditions became larger over time, reflecting a condition-by-trial interaction (*X*^*2*^(1) = 4.38, *p* = .04). The interaction between age and trial reached significance (*X*^*2*^(1) = 6.89, *p* = .009), with children’s controllability estimates increasing over the course of the task and adults’ decreasing, reflecting an initial bias toward controllability beliefs that becomes more accurate across trials. Moreover, these arbitrator values became more divergent across controllability conditions in older compared to younger individuals and increasingly so across the task (age-by-condition: *X*^*2*^(1) = 6.01, *p* = .01; age-by-condition-by-trial: *X*^*2*^(1) = 5.85, *p* = .02), capturing that adults are better at assessing the true degree of environmental controllability. No other effects reached significance (*p*’s > .3). As parameter estimates were derived by fitting the model to participants’ state predictions, we used the condition predictions, which served as an entirely independent dataset, to assess how well binary choices about the current condition were predicted by the Task Set model. A logistic regression revealed that model-derived estimates of controllability, indexed by ⍵, were strongly related to condition predictions (one-sample t-test on individual regression slopes: *t*(89) = 8.57, *p* < 0.001), and increasingly so with age (correlation between slopes and age: rho = 0.37, *p* < 0.001).

As working memory mediated the age-related improvement in assessment of controllability, we next tested whether working memory related to individuals’ use of the simpler Spectator model, which requires holding in mind only the transitions between states, versus the other, more complex models that additionally take into account the consequences of actions, and thus require tracking a greater number of state transitions. We hypothesized that individuals with better working memory might show greater use of these more complex mental models of their environment, which would be reflected by a larger difference in quality of fit (*Δ* BIC) between the simpler and more complex models. Indeed, the difference in model fit between the Spectator and Task Set models increased with age (*β* = .49, SE = .1, *t* = 5.12 *p* < .001). A formal mediation analysis revealed working memory as a significant partial mediator between age and *Δ* BIC for the Spectator and Task Set models (standardized indirect effect: .06, 95% confidence interval: [.004: .16], *p* = .028; standardized direct effect: .43, 95% confidence interval: [.24: .62], *p* < .001; Fig 3E). Working memory also showed a significant partial mediation of the relation between age and *Δ* BIC between the Spectator model and all other models that incorporate the consequences of actions (i.e., the Learned Transition Structure and Actor models; see S2 Appendix). This suggests that working memory contributes to age-related increases in the use of mental models that incorporate actions, and their consequences, into assessments of control.

**Fig 4:**
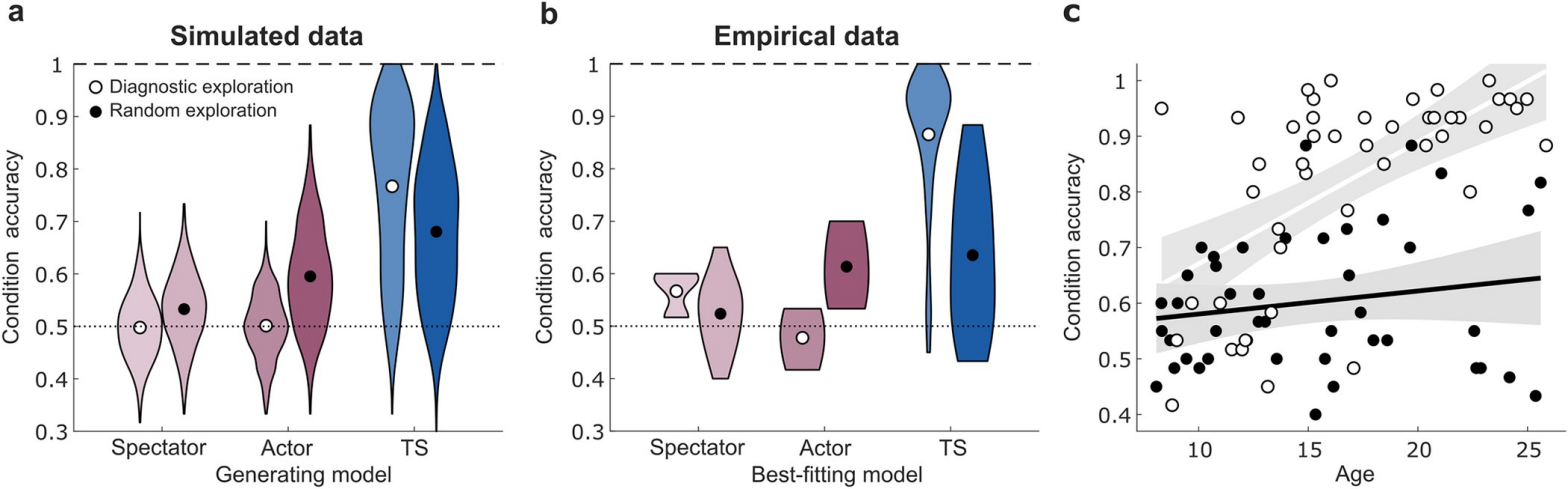
Simulated and empirical data for condition prediction accuracy as a function of diagnostic interventions. (a) Simulations reveal that consistently selecting the diagnostic exploratory choice is only beneficial for the Task Set (TS) model. Random exploration yields better controllability assessments for all other models. (b) A median-split of participants based on their proportion of diagnostic interventions closely matches model predictions and shows a unique performance benefit of diagnostic choices for individuals best fit by the Task Set model. Given that only two participants were best fit by the Learned Transition Structure model, it was not included in these analyses. (c) A median-split analysis revealed that age-related improvements in condition prediction accuracy were driven largely by participants who made more diagnostic choices (open circles) whereas no significant age-related improvement was evident for participants who explored more randomly (filled circles). Shaded areas represent 95% confidence intervals. Abbreviations: TS: Task Set.

Whereas older individuals used task structure knowledge to make more informative interventions on exploratory trials, younger individuals exhibited greater randomness in their exploratory choices. To investigate whether the random exploration evident in children constitutes a resource-rational way of generating evidence for outcome prediction when inferential abilities are not as robust, we simulated data for each of the four models, varying whether interventions were entirely diagnostic or random. The simulations show that consistently selecting the diagnostic exploratory choice only facilitated controllability assessment for the Task Set model (Fig 4A). For the other models that do not implement inferential strategies, random interventions yielded better performance. Empirical data matched these qualitative trends, such that individuals best fit by the Task Set model who made more diagnostic interventions (as determined by a median-split on the proportion of diagnostic choices across all participants) exhibited greater accuracy on condition predictions (Fig 4B). In contrast, individuals best fit by any of the other three non-inferential models showed better performance if they explored more randomly. Thus, random exploration may provide younger learners with a compensatory mechanism for predicting outcomes when working memory capacity is not yet sufficient to represent and make inferences based on the full task transition structure.

We reasoned that if age-related improvement in controllability assessment stemmed from the synergistic development of diagnostic interventions and inferential processes, then this improvement should be more pronounced in individuals who selected the diagnostic exploratory choice more frequently. Accordingly, we found that condition prediction accuracy increased with age in the half of participants (median-split) who more frequently made diagnostic interventions (71+/-14% of diagnostic choices, r = 0.61, *p* < .001). In contrast, for the half of participants who explored more randomly or frequently selected the uninformative choice (40+/-16% of diagnostic choices), there was no age-related improvement in condition predictions (r = 0.18, *p* = 0.25, difference in correlation coefficients: z = 2.43, *p* = 0.008, Fig 4C). This pattern of findings was qualitatively similar for state prediction accuracy (see S4 Fig).

### Explicit knowledge

Our model comparison results suggest that many participants use the task rules to ascertain the current condition by making diagnostic choices and that this may become the dominant strategy as individuals grow older. Given that such explicit knowledge about the task transition structure might account for age-related improvements in accuracy, following the game we probed participants about their explicit knowledge of the rules by which the two pilots flew. As perfect knowledge of the transition structure was quite common (82% of participants), we performed a chi-squared test to assess age differences in perfect explicit knowledge of the task transition structure between children, adolescents, and adults. This analysis revealed a significant difference in the distribution of participants who accurately reported all six transitions (*X*^*2*^(2) = 6.537, *p* = 0.038). Additional pairwise chi-squared tests confirmed that the proportion of children (*X*^*2*^(1) = 6.405, *p* = 0.011) and adolescents (*X*^*2*^(1) = 5.192, *p* = 0.023) who accurately answered all six questions significantly differed from adults, revealing that a greater proportion of adults had perfect knowledge of the task structure. However, no difference was observed between the proportion of children and adolescents who responded correctly to all the questions (*X*^*2*^(1) = .089, *p* = 0.766). Thus, explicit knowledge of the task rules is more prevalent in adults than younger individuals, supporting the increased use of a hypothesis-testing strategy with age.

## Discussion

In this study, we investigated developmental changes in the ability to detect the controllability of the environment and select informative interventions that reveal its causal structure. Children, adolescents, and adults completed a task that covertly alternated between a controllable and uncontrollable condition whose causal structure could be discovered through exploratory interventions. Throughout the task, participants were frequently probed about their beliefs of environmental controllability, allowing us to study controllability detection with high temporal resolution. Children tended to make more random exploratory choices, whereas older participants made more interventions that provided diagnostic evidence of the current condition. We found that children distinguished between controllable and uncontrollable conditions with a high degree of accuracy, however accuracy of these assessments improved with age into adulthood. Across all ages, controllability assessments were more accurate in the controllable versus the uncontrollable condition. Computational modeling showed that age-related improvements in controllability assessment stemmed from an increased ability to represent the structure of contingencies between states and actions and to use that knowledge to infer the current state of control by making informative interventions. Working memory contributed to this developmental transition by supporting older individuals’ use of more complex mental representations of the task structure. In contrast, younger individuals’ greater reliance on random exploration may be a resource-rational strategy for learning environmental contingencies when working memory abilities are still developing [68,69]. Taken together, our findings suggest that a qualitative shift toward greater use of inferential reasoning may lead to more accurate controllability assessments with age, which may facilitate better detection of opportunities to engage in goal-directed action across development.

Consistent with an extensive literature suggesting the biological and psychological importance of environmental control [12,17,70], we found that participants of all ages were more prone to infer control rather than the lack thereof, suggesting a bias toward beliefs of controllability. Such a bias may indeed be beneficial. Beliefs about controllability are proposed to generalize to novel situations where the environmental structure is unknown [5,6,71]. Generalizing controllability beliefs can promote active exploratory behavior and cognitive engagement, which facilitate the discovery of action-outcome affordances in novel environments [5,6]. In contrast, as famously demonstrated in early studies of learned helplessness [70], experiences of uncontrollability decrease exploration, attention [72], and contingency learning in novel contexts [73], perhaps reflecting reduced engagement of goal-directed learning processes when past experience has suggested that they are likely to be ineffective. Such generalization effects have been proposed to reflect an adaptive calibration of cognitive effort and behavioral strategies to the degree of agency afforded by an environment [6], a process that can occur over both local and developmental timescales [3,74]. Such a bias toward controllability beliefs early in life [75] may be particularly advantageous as it reduces the potential opportunity costs of curtailing exploration and goal-directed learning when uncertainty about the true degree of environmental controllability is still high.

Whereas adults appeared to use a mental model of the task structure to promote informative interventions and accurate causal inferences, children and adolescents showed more difficulty using instructed information to guide their exploratory choices and controllability assessments. At the start of the task, participants were taught the action-outcome contingencies that applied in the controllable condition and the sequence of transitions that occurred in the uncontrollable condition. Prior work suggests that adults can rapidly learn to adjust their behavior according to instructed tasks rules [76]. In contrast, younger individuals, who may not yet be adept at using mental representations of a task’s structure to inform choices [36,37,55], tend to favor learning from experience over the use of instructed information [56,77]. Children’s near-chance selection of the diagnostic exploratory choice, as well as their broad exploration of the potential actions available in each state, is consistent with a vast literature highlighting a shift from greater reliance on exploration to exploitation with age [45–47,49,78,79], and may reflect a greater propensity of younger individuals to estimate controllability from the statistics of experienced outcomes, rather than using task structure knowledge to perform diagnostic hypothesis tests. Children’s greater reliance on random exploration has previously been shown to benefit learning [48] and may reflect a resource-rational strategy in younger individuals whose working memory abilities are not as robust [68,69]. An open question is whether the observed age differences in the accurate assessment of controllability and explicit knowledge about the task structure might be mitigated in a context where the statistical regularities between states and actions must be discovered through experience, in the absence of explicit instruction. Although much of the learning in this task occurs in the absence of feedback, another avenue for future research is whether age-related differences in processing positive and negative outcomes, as previous work suggests [80,81], or in confidence about one’s assessment of the degree of environmental controllability may contribute to developmental changes in accurately assessing control.

Under multiple theoretical accounts, estimation of environmental controllability acts as a meta-level learning process that can be used to adapt one’s cognition and behavior to situational demands. Controllability assessments allow for the appropriate assignment of credit to oneself or to external causes when learning the value of actions [54,82] and amplifies learning signals for agentic choices [22]. Like the ability to accurately detect the state of environmental controllability assessed here, such use of controllability inferences to inform reinforcement learning has been found to increase with age [55]. The rational allocation of cognitive control to support goal-directed behavior is also proposed to depend on assessments of environmental controllability [1,2,83,84]. In line with theoretical proposals that deliberative action selection should be preferentially engaged in controllable environments [5,6], adults can use dynamic estimates of controllability to arbitrate between using learned action-outcome contingencies to achieve goals versus simpler reflexive strategies that assume no contingency between actions and outcomes [2,85]. Our present findings that controllability assessments improve into young adulthood suggest that children may similarly be less adept at adjusting their reward-motivated behavior according to the degree of environmental controllability. However, future work is necessary to better understand whether arbitration between these value-based learning systems across controllable and uncontrollable contexts changes with age.

As our study is cross-sectional in nature and our sample size is moderate, caution should be used when interpreting the results of our working memory mediation analyses. Previous work has shown that mediation analyses performed on cross-sectional samples may not always correspond to the dynamics of an underlying developmental process that unfolds across time [86,87]. Future studies that adopt a longitudinal approach are needed to better characterize the contribution of working memory development to the ability to accurately assess environmental controllability from childhood to adulthood. Moreover, studies that extend this age range into older adulthood might assess whether known declines in working memory ability in older adults compromise their ability to use inferential strategies to assess controllability.

The ability to determine when one’s actions are consequential provides foundational knowledge about the structure of the environment that organizes an individual’s learning and decision making. The present study demonstrated that from childhood to adulthood, individuals were able to detect dynamic changes in environmental controllability, and that the accuracy of these assessments improved with age. Across development, we also found that assessments were biased toward beliefs of control, a tendency with potentially beneficial consequences for learning and mental health. Children, adolescents and adults exhibited a synergistic coupling between their exploratory behaviors and the nature of their learning strategies, which was dependent upon the cognitive resources available at their respective developmental stages. Collectively, these findings reveal a qualitative shift across development in the underlying strategies through which individuals recognize when actions can be leveraged for their benefit.

## Supporting information

S1 Fig Models of more complex mental representations show improvements in fit with age. (a-b) Cognitive models that incorporate the effects of action selection (Actor model; a) and explicit knowledge of the task rules (Task Set model; b) provide a better fit with increasing age, as measured by relative difference in Bayesian Information Criterion (BIC). The line of best fit is shown, along with 95% confidence intervals. The following abbreviations are used: LTS: Learned Transition Structure model; TS: Task Set model.
(TIF)

S2 Fig Parameter distributions and recovery analyses. (**a**) Empirical distribution of best-fitting parameters in their native space (i.e before transformation constraining them to specific ranges). The blue lines denote the posterior Gaussian distributions from which parameters were randomly drawn for the simulations. The black dashed lines denote the prior Gaussian distributions used as priors in the model fitting routine. (**b**) The model recovery analysis demonstrated the very high recoverability of candidate models. The lowest recovery rate, found for the Learned Transition Structure model (LTS), was still superior to 95%. (**c**) The parameter recovery analysis performed for the best fitting model (TS, Task Set) demonstrated that all parameters were identifiable. For the least identifiable parameter, “slope Ω”, the correlation coefficient between generating and recovered values was still superior to 0.62. The learning rate and inverse temperature parameters, which correlated with age (see Main text), were the most recoverable parameters, with correlation coefficients of 0.85 and 0.94, respectively. **(d)** Model and **(e)** parameter recoverability are shown using parameter estimates drawn from distributions fit only to children’s data (8–12 years old).
(TIF)

S3 Fig Parameter estimates from the Task Set model by age. (a-d) Parameter estimates from the Task Set model, which provided the best fit at the group level, are plotted across age. The line of best fit is shown, and shaded error bars represent 95% confidence intervals.
(TIF)

S4 Fig Simulated and empirical data for state prediction accuracy as a function of diagnostic interventions showing effects qualitatively similar to those reported in Fig 4. (a) Diagnostic exploratory choice is only beneficial for the Task Set model. Random exploration yields better controllability assessments for all other models. (b) A median-split of participants based on their proportion of diagnostic interventions closely matches model predictions and shows a unique performance benefit of diagnostic choices for individuals best fit by the Task Set model. Given that only two participants were best fit by the Learned Transition Structure model, it was not included in these analyses. (c) A median-split analysis showed that age-related improvements in performance were more salient in the group of participants who made more diagnostic choices (open circles) than in the group who explored more randomly (filled circles). However, the difference between the two slopes was not significantly significant (z = 0.84, p = 0.2). Shaded areas represent 95% confidence intervals. Abbreviations: TS: Task Set.
(TIF)

S1 Appendix Additional behavioral analyses and full results for mixed-effect models. Table A. State prediction accuracy with working memory Table B. State prediction accuracy without working memory Table C. Condition prediction accuracy with working memory Table D. Condition prediction accuracy without working memory Table E. Diagnostic choice during exploratory trials
(DOCX)

S2 Appendix Computational modeling specifications and analyses. Table A. Model comparison testing age vs. age^2^ for model-derived estimates
(DOCX)
