# Supplementary figures and images for: Developmental shifts in computations used to detect environmental controllability
Source: PLoS Comput Biol. 2022 Jun 1;18(6):e1010120. doi: 10.1371/journal.pcbi.1010120 (PMC9191713; doi:10.1371/journal.pcbi.1010120)

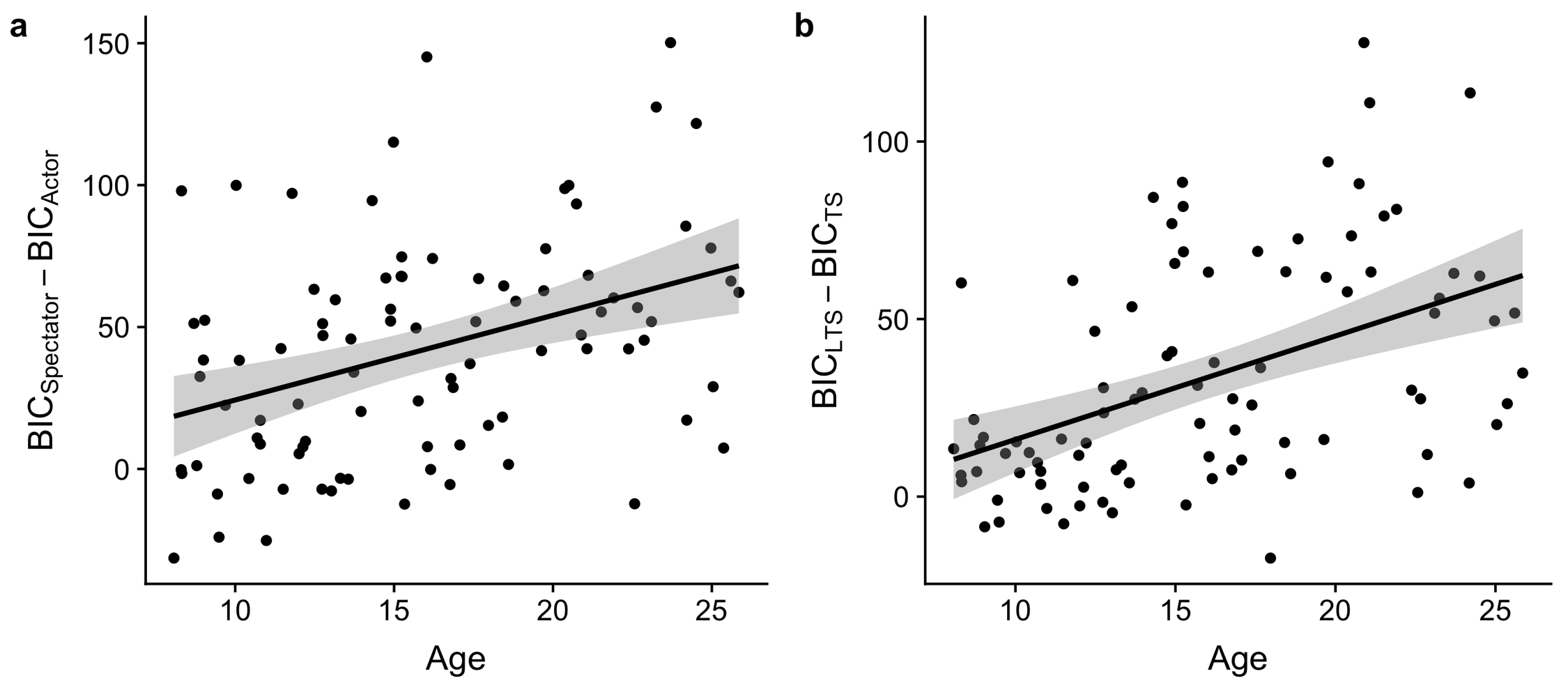

Supplement: S1 Fig — (a-b) Cognitive models that incorporate the effects of action selection (Actor model; a) and explicit knowledge of the task rules (Task Set model; b) provide a better fit with increasing age, as measured by relative difference in Bayesian Information Criterion (BIC). The line of best fit is shown, along with 95% confidence intervals. The following abbreviations are used: LTS: Learned Transition Structure model; TS: Task Set model. (TIF) [file pcbi.1010120.s001.tif]

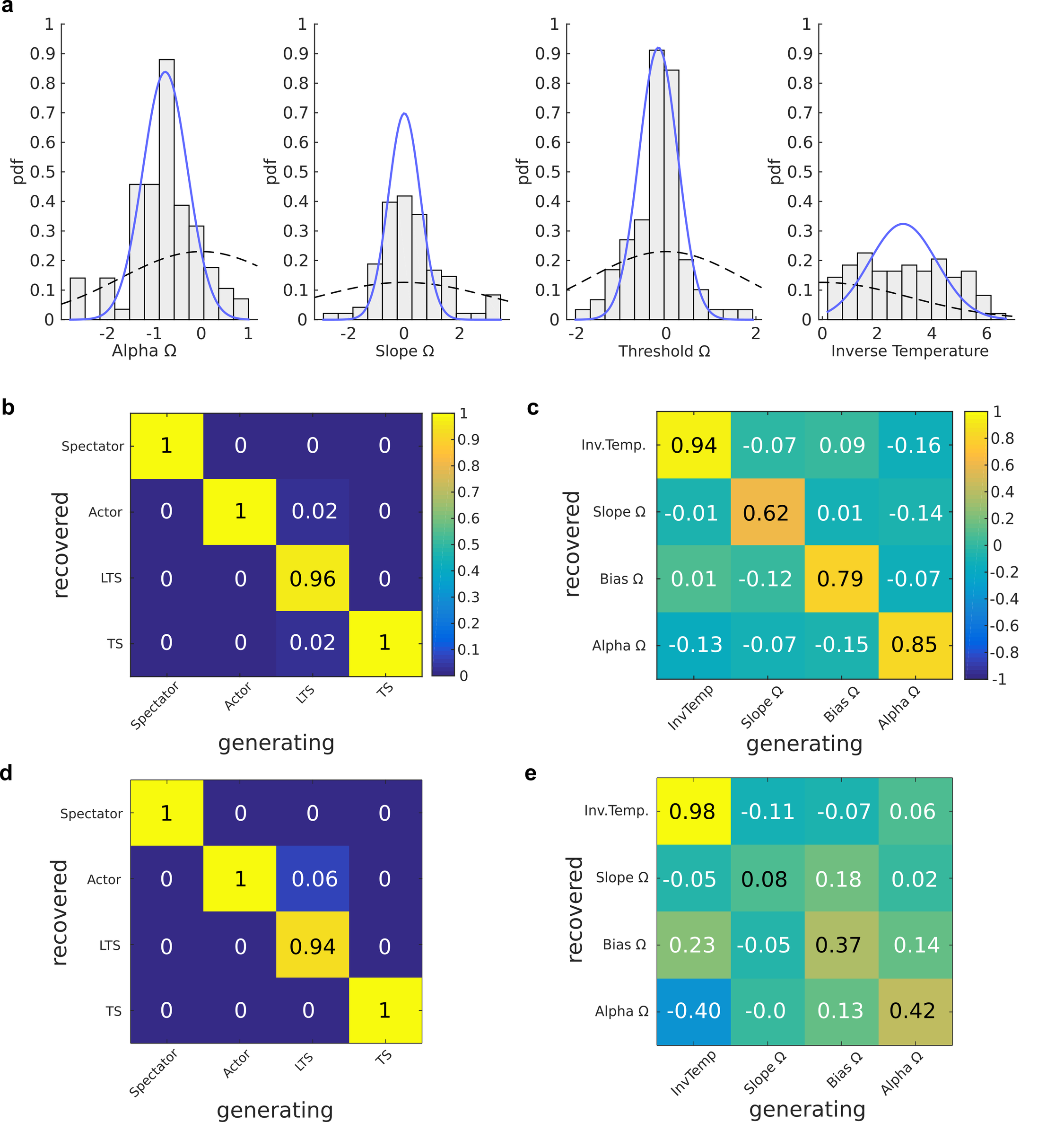

Supplement: S2 Fig — (a) Empirical distribution of best-fitting parameters in their native space (i.e before transformation constraining them to specific ranges). The blue lines denote the posterior Gaussian distributions from which parameters were randomly drawn for the simulations. The black dashed lines denote the prior Gaussian distributions used as priors in the model fitting routine. (b) The model recovery analysis demonstrated the very high recoverability of candidate models. The lowest recovery rate, found for the Learned Transition Structure model (LTS), was still superior to 95%. (c) The parameter recovery analysis performed for the best fitting model (TS, Task Set) demonstrated that all parameters were identifiable. For the least identifiable parameter, “slope Ω”, the correlation coefficient between generating and recovered values was still superior to 0.62. The learning rate and inverse temperature parameters, which correlated with age (see Main text), were the most recoverable parameters, with correlation coefficients of 0.85 and 0.94, respectively. (d) Model and (e) parameter recoverability are shown using parameter estimates drawn from distributions fit only to children’s data (8–12 years old). (TIF) [file pcbi.1010120.s002.tif]

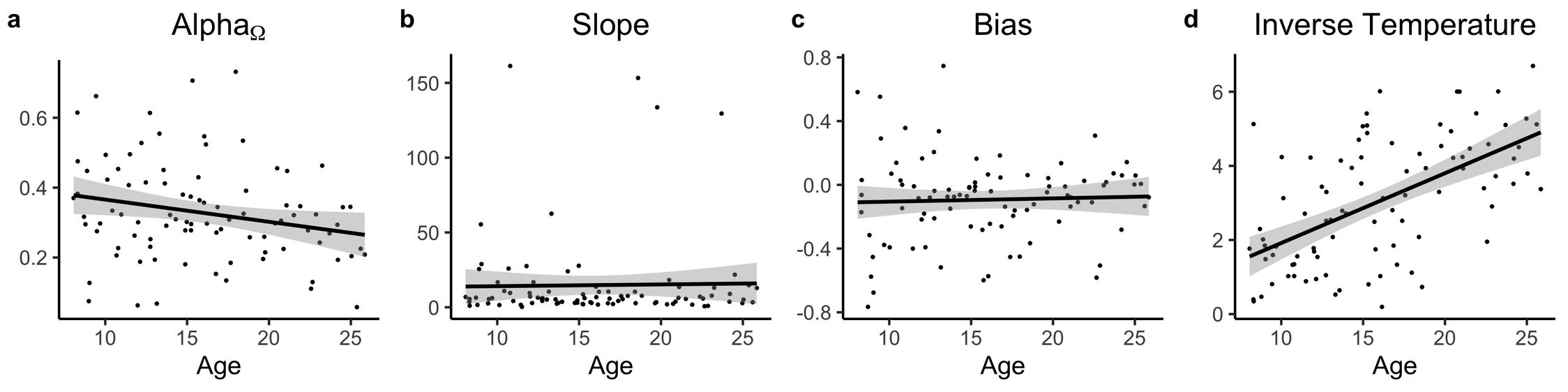

Supplement: S3 Fig — (a-d) Parameter estimates from the Task Set model, which provided the best fit at the group level, are plotted across age. The line of best fit is shown, and shaded error bars represent 95% confidence intervals. (TIF) [file pcbi.1010120.s003.tif]

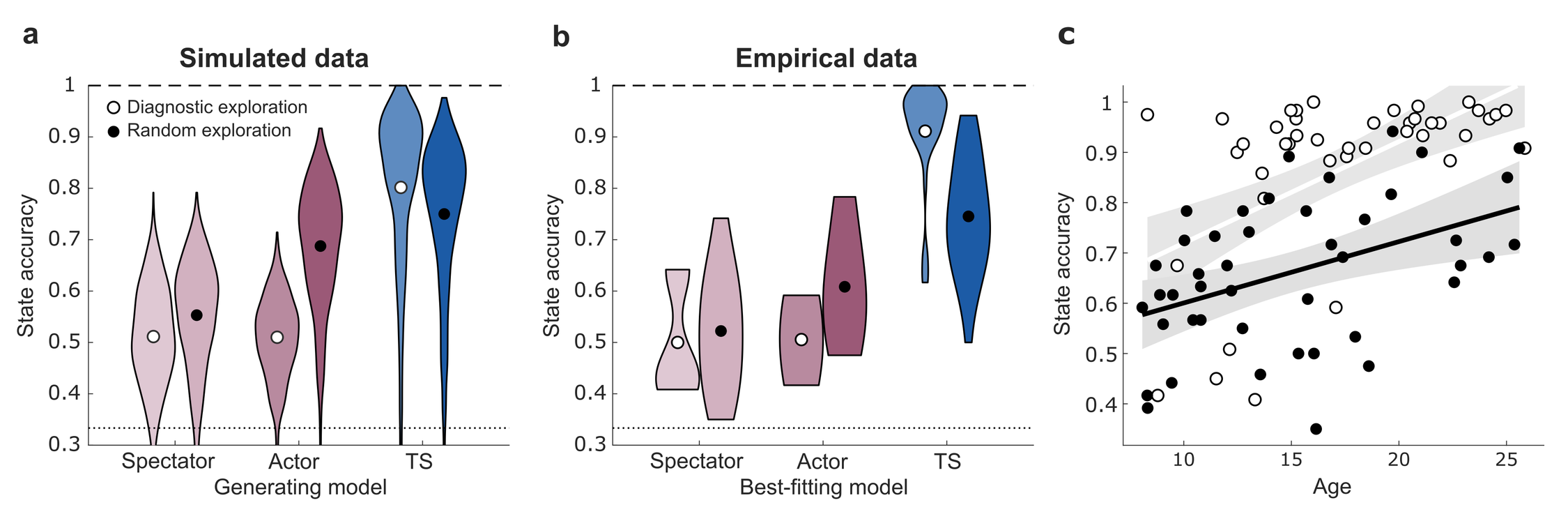

Supplement: S4 Fig — (a) Diagnostic exploratory choice is only beneficial for the Task Set model. Random exploration yields better controllability assessments for all other models. (b) A median-split of participants based on their proportion of diagnostic interventions closely matches model predictions and shows a unique performance benefit of diagnostic choices for individuals best fit by the Task Set model. Given that only two participants were best fit by the Learned Transition Structure model, it was not included in these analyses. (c) A median-split analysis showed that age-related improvements in performance were more salient in the group of participants who made more diagnostic choices (open circles) than in the group who explored more randomly (filled circles). However, the difference between the two slopes was not significantly significant (z = 0.84, p = 0.2). Shaded areas represent 95% confidence intervals. Abbreviations: TS: Task Set. (TIF) [file pcbi.1010120.s004.tif]
