# Supplementary material for: Developmental shifts in computations used to detect environmental controllability
Source: PLoS Comput Biol. 2022 Jun 1;18(6):e1010120. doi: 10.1371/journal.pcbi.1010120 (PMC9191713; doi:10.1371/journal.pcbi.1010120)
Supplement: S1 Appendix — Table A. State prediction accuracy with working memory Table B. State prediction accuracy without working memory Table C. Condition prediction accuracy with working memory Table D. Condition prediction accuracy without working memory Table E. Diagnostic choice during exploratory trials (DOCX) [file pcbi.1010120.s005.docx]

Developmental shifts in computations

used to detect environmental controllability

Hillary A. Raab, Careen Foord, Romain Ligneul, & Catherine A. Hartley

**S1 Appendix.** Additional behavioral analyses and full results for mixed-effect models.

**No relationship between age and WASI**

To ensure that general reasoning ability did not co-vary with age in our sample, we performed a linear regression to determine the relationship between age and scores on the WASI. A model including age alone, rather than one including age and age-squared, fit best (*F*(1,87) = .002, *p* = .963). Age was not a significant predictor of normed WASI scores (*β* = -1.625, s.e. = 1.48, *t*(88) = -1.098, *p* = .275, Cohen’s 𝑓^2^ = .014), indicating that any variation in performance couldn’t be attributed to differences in reasoning ability.

**No difference in state or condition prediction accuracy based on differences in probabilistic state transitions**

As the transition probabilities between states differed across the four runs of the task (the first and third run had 90% probabilistic transitions while the second and fourth run had 80% probabilistic transitions), we ran a mixed-effects logistic regression including age and transition probability type to examine whether performance on state prediction accuracy differed based on transition probabilities. Performance did not differ between the runs with 80% versus 90% transition probabilities (transition probability: *𝛸^2^*(1) = 1.15, *p* = .28, age: *𝛸^2^*(1) = 26.55, *p* < .0001, age*transition probability: *𝛸^2^*(1) = 1.13, *p* = .29). We repeated this analysis for condition prediction accuracy. As the maximal model didn’t converge, we only included a subject-specific random intercept in the model. Condition prediction accuracy showed no difference based on transition probabilities (transition probability: *𝛸^2^*(1) = .17, *p* = .68, age: *𝛸^2^*(1) = 18.31, *p* < .0001, age*transition probability: *𝛸^2^*(1) = .04, *p* = .84).

**High correspondence between state and condition predictions**

State predictions served as an indirect measure of controllability, whereas condition predictions served as a direct assessment of controllability. Thus, we examined the correspondence between these two assessments of environmental controllability. We ran a linear regression including average accuracy on state predictions as a predictor of average accuracy on condition predictions. The model that included age alone, rather than the addition of an age-squared term, provided a better fit (*F*(2) = 1.17, *p* = .316). Higher accuracy on condition predictions was associated with better performance on state predictions and age (state predictions: *β* = .958, s.e. = .071, *t*(86) = 13.41, *p* < .0001; age: *β* = -.119, s.e. = .057, *t*(86) = -2.089, *p* = .05). Moreover, state predictions were a slightly better indicator of performance on condition predictions in older as compared to younger individuals (*β* = .142, s.e. = .071, *t*(86) = 1.996, *p* = .049).

**Significant effect of trials since reversal on state but not condition prediction accuracy**

As performance may worsen immediately following the reversal of conditions, we conducted a mixed-effects logistic regression including the number of state prediction trials since reversal, age, and condition on state prediction accuracy. The full model that included condition and trials since reversal in the random effect structure did not converge. Thus, we removed trials since reversal from the random effects. The model that included age alone provided a better fit than the addition of age-squared (*𝛸^2^*(4) = 3.08, *p* = .545). We found a significant effect of trials since reversal on state prediction accuracy (*𝛸^2^*(1) = 4.02, *p* = .04). In addition, age and condition were also significant main effects (age: *𝛸^2^*(1) = 26.44, *p* < .0001; condition: *𝛸^2^*(1) = 18.57, *p* < .0001). There were no significant interactions (*p*’s > .2). We repeated this analysis for condition prediction accuracy. As the maximal model didn’t converge, we only included condition as a random effect in the model. Again, the model that included age alone provided a better fit than the addition of age-squared (*𝛸^2^*(4) = 2.0, *p* = .736). We found that trials since reversal did not relate to condition prediction accuracy (*𝛸^2^*(1) = .27, *p* = .60). However, age and condition were significant (age: *𝛸^2^*(1) = 18.60, *p* < .0001; condition: *𝛸^2^*(1) = 7.84, *p* = .005). There were no significant interactions (*p*’s > .2).

**Response times slower in controllable condition for state but not condition predictions**

As response times may also reflect differences in controllability assessments, we conducted a mixed-effect linear regression examining the effects of age, condition, and trials since reversal on response time on state prediction trials. We log-transformed response times prior to conducting the analysis. We removed the random effect of trials since reversal as the maximal model did not converge. The inclusion of an age-squared term as a predictor in the model did not improve the fit (*𝛸^2^*(4) = 2.3, *p* = .682). Response times differed as a function of condition, with participants responding faster in the controllable condition (*𝛸^2^*(1) = 5.4, *p* = .02). No other main effects or interactions reached significance (*p*’s > .05).

We repeated this analysis for condition prediction accuracy. Again, we log-transformed response times and removed trials since reversal from the random slope structure as the maximal model did not converge. The model including only linear age provided a better fit than the addition of an age-squared term (*𝛸^2^*(4) = 6.51, *p* = .164). The effect of condition nearly reached significance, with participants slower in the uncontrollable condition (*𝛸^2^*(1) = 3.74, *p* = .05). Participants became significantly faster with more trials since the last reversal (*𝛸^2^*(1) = 5.48, *p* = .02) and with age (*𝛸^2^*(1) = 30.56, *p* < .001).

**Mixed-effects models: State prediction accuracy with and without working memory**

We sought to examine how condition, state prediction trial number, proportion optimal choice on every six exploratory trials, working memory (only for one of the models), and age influence state prediction accuracy. As working memory performance was not collected on six individuals, we performed two generalized mixed-effects logistic regressions, one that included working memory as a predictor (n = 84) and one that did not (n = 90). We used sum-to-zero contrasts for the categorical variable *condition*, which reflects deviation from the grand mean. The uncontrollable condition was coded as 0, and the controllable condition was coded as 1. The goal was for these models to have identical structure, except for the working memory term. Thus, we selected the most complex random effect structure that converged for both models. The model included subject-specific random intercepts and random slopes for condition, trial number, and proportion of optimal exploratory choices. As the maximal model did not converge, we removed the correlation between the random slopes and intercepts. Due to convergence issues, we also removed the two-way interaction between optimal choice and trial number and the three-way interaction in the random effect structure.

Full model: Response accuracy ~ condition * age * trial number * (proportion diagnostic choice + working memory) + (1 + (proportion diagnostic choice + trial number) * condition || SubjID)

For the mixed-effects model without working memory, a statistical model that included the addition of an age-squared term did not provide a better fit than age alone (*𝛸^2^*(8) = 5.46, *p* = .707). Age, condition, trial number, and optimal exploratory choice were the only main effects to reach significance (age: *𝛸^2^*(1) = 31.92, *p* < .0001; condition: *𝛸^2^*(1) = 15.81, *p* < .0001; trial: *𝛸^2^*(1) = 9.81, *p* = .002; optimal choice: *𝛸^2^*(1) = 8.3, *p* = .004), just as in the analysis that included working memory. Condition-by-optimal choice and age-by-optimal choice also remained significant (condition-by-optimal choice: *𝛸^2^*(1) = 6.95, *p* = .008; age-by-optimal choice: *𝛸^2^*(1) = 7.75, *p* = .005). No effects that weren’t significant became so.

Full model: Response accuracy ~ condition * age * trial number * proportion diagnostic choice + (1 + (proportion diagnostic choice + trial number) * condition || SubjID)

Table A. State prediction accuracy with working memory

| Predictor | Regression Coefficient (*SE*) | *χ^2^* | *p* |
| --- | --- | --- | --- |
| **Intercept** | **1.65 (.12)** |  |  |
| **Condition** | **-.21 (.06)** | **12.71** | **.0004** |
| **Age** | **.77 (.12)** | **32.88** | **< .0001** |
| **Trial number** | **.14 (.04)** | **11.35** | **.0008** |
| **Optimal choice** | **.15 (.06)** | **7.30** | **.007** |
| Working memory | .22 (.12) | 3.10 | .08 |
| Condition x Age | .01 (.06) | .01 | .91 |
| Condition x Trial number | .05 (.04) | 1.69 | .19 |
| **Condition x Optimal choice** | **.11 (.05)** | **5.07** | **.02** |
| Condition x Working memory | -.04 (.06) | .51 | .47 |
| Age x Trial Number | -.02 (.04) | .16 | .69 |
| **Age x Optimal choice** | **.16 (.06)** | **7.82** | **.005** |
| Age x Working memory | -.16 (.13) | 1.35 | .25 |
| Trial number x Optimal choice | .02 (.03) | .27 | .61 |
| Trial number x Working memory | -.02 (.04) | .14 | .71 |
| Condition x Age x Trial number | .07 (.04) | 2.53 | .11 |
| Condition x Age x Optimal choice | .06 (.05) | 1.5 | .22 |
| Condition x Age x Working memory | -.04 (.06) | .39 | .53 |
| Condition x Trial number x Optimal choice | .01 (.03) | 0.06 | .8 |
| Condition x Trial number x Working memory | -.02 (.04) | .29 | .59 |
| Age x Trial number x Optimal choice | -.01 (.03) | .04 | .84 |
| Age x Trial number x Working memory | .02 (.05) | .26 | .61 |
| Condition x Age x Trial number x Optimal choice | .01 (.03) | .06 | LJFJ .81 |
| Condition x Age x Trial number x Working memory | .01 (0.05) | .06 | .8 |

Table B. State prediction accuracy without working memory

| Predictor | Regression Coefficient (*SE*) | *χ^2^* | *p* |
| --- | --- | --- | --- |
| Intercept | 1.65 (.12) |  |  |
| **Condition** | **-.23 (.06)** | **15.81** | **< .0001** |
| **Age** | **.76 (.12)** | **31.92** | **< .0001** |
| **Trial number** | **.13 (.04)** | **9.81** | **.002** |
| **Optimal choice** | **.15 (.05)** | **8.3** | **.004** |
| Condition x Age | 0 (.06) | 0 | .96 |
| Condition x Trial number | .06 (.04) | 2.15 | .14 |
| **Condition x Optimal choice** | **.12 (.04)** | **6.95** | **.008** |
| Age x Trial number | -.03 (.04) | .37 | .54 |
| **Age x Optimal choice** | **.15 (.05)** | **7.75** | **.005** |
| Trial number x Optimal choice | .01 (.03) | .07 | .8 |
| Condition x Age x Trial number | .07 (.04) | 2.62 | .11 |
| Condition x Age x Optimal choice | .06 (.04) | 1.58 | .21 |
| Condition x Trial number x Optimal choice | .02 (.03) | .39 | .53 |
| Age x Trial number x Optimal choice | -.01 (.03) | .17 | .68 |
| Condition x Age x Trial number x Optimal choice | .01 (.03) | .08 | .77 |

**Mixed-effects models: Condition prediction accuracy with and without working memory**

We sought to examine how condition, condition prediction trial number, proportion optimal choice on every six exploratory trials, working memory (only for one of the models), and age influence condition prediction accuracy. As working memory performance was not collected on six individuals, we performed two generalized mixed-effects logistic regressions, one that included working memory as a predictor (n = 84) and one that did not (n = 90). We used sum-to-zero contrasts for the categorical variable *condition*, which reflects deviation from the grand mean. The uncontrollable condition was coded as 0, and the controllable condition was coded as 1. The goal was for these models to have identical structure, except for the working memory term. Thus, we selected the most complex random effect structure that converged for both models. We started with the maximal random effect structure, which did not converge. We then removed the correlation between random intercepts and slopes. As this model still did not converge, we removed the two-way interaction between optimal choice and trial number and the three-way interaction in the random effect structure.

Full model: Response accuracy ~ condition * age * trial number * (proportion diagnostic choice + working memory) + (1 + (proportion diagnostic choice + trial number) * condition || SubjID)

As in the analysis in the main text, a statistical model that included the addition of an age-squared term did not provide a better fit than age alone for the set of models without working memory (*𝛸^2^*(8) = 2.87, *p* = .942). All significant effects remained so, and no additional effects became significant. See Table D in S1 Appendix for results.

Full model: Response accuracy ~ condition * age * trial number * proportion diagnostic choice + (1 + (proportion diagnostic choice + trial number) * condition || SubjID)

Table C. Condition prediction accuracy with working memory

| Predictor | Regression Coefficient (*SE*) | *χ^2^* | *p* |
| --- | --- | --- | --- |
| **Intercept** | **1.62 (.13)** |  |  |
| **Condition** | **-.17 (.05)** | **9.38** | **.002** |
| **Age** | **.71 (.13)** | **26.95** | **< .0001** |
| **Trial number** | **.11 (.05)** | **4.86** | **.03** |
| **Optimal choice** | **.3 (.07)** | **17.32** | **< .0001** |
| **Working memory** | **.25 (.13)** | **3.73** | **.05** |
| Condition x Age | -.07 (.06) | 1.56 | .21 |
| Condition x Trial number | -.02 (.05) | .19 | .66 |
| Condition x Optimal choice | .07 (.07) | 1.21 | .27 |
| Condition x Working memory | .01 (.06) | .02 | .89 |
| Age x Trial Number | -.05 (.05) | .83 | .36 |
| **Age x Optimal choice** | **.29 (.07)** | **16.78** | **< .0001** |
| Age x Working memory | -.08 (.14) | .28 | .59 |
| Trial number x Optimal choice | -.04 (.04) | .85 | .36 |
| Trial number x Working memory | -.02 (.05) | .24 | .62 |
| Condition x Age x Trial number | .02 (.05) | .2 | .65 |
| Condition x Age x Optimal choice | .07 (.06) | 1.27 | .26 |
| Condition x Age x Working memory | .01 (.06) | .04 | .84 |
| Condition x Trial number x Optimal choice | .04 (.05) | .78 | .38 |
| Condition x Trial number x Working memory | -.07 (.05) | 2.04 | .15 |
| Age x Trial number x Optimal choice | -.01 (.04) | .11 | .74 |
| Age x Trial number x Working memory | -.04 (.05) | .59 | .44 |
| Condition x Age x Trial number x Optimal choice | .02 (.04) | .31 | .58 |
| Condition x Age x Trial number x Working memory | -.07 (.06) | 1.6 | .21 |

Table D. Condition prediction accuracy without working memory

| Predictor | Regression Coefficient (*SE*) | *χ^2^* | *p* |
| --- | --- | --- | --- |
| **Intercept** | **1.61 (.12)** |  |  |
| **Condition** | **-.17 (.05)** | **11.02** | **.0009** |
| **Age** | **.7 (.13)** | **26.9** | **< .0001** |
| **Trial number** | **.11 (.04)** | **6.07** | **.01** |
| **Optimal choice** | **.32 (.07)** | **21.81** | **< .0001** |
| Condition x Age | -.08 (.05) | 1.88 | .17 |
| Condition x Trial number | -.01 (.05) | .07 | .79 |
| Condition x Optimal choice | .07 (.06) | 1.49 | .22 |
| Age x Trial Number | -.04 (.05) | .72 | .4 |
| **Age x Optimal choice** | **.29 (.07)** | **18.07** | **< .0001** |
| Trial number x Optimal choice | -.03 (.04) | .52 | .47 |
| Condition x Age x Trial number | .03 (.05) | .36 | .55 |
| Condition x Age x Optimal choice | .06 (.06) | 1.03 | .31 |
| Condition x Trial number x Optimal choice | .03 (.04) | .42 | .52 |
| Age x Trial number x Optimal choice | -.02 (.04) | .16 | .69 |
| Condition x Age x Trial number x Optimal choice | .02 (.04) | .22 | .64 |

**Mixed-effects model: Diagnostic exploratory choice**

We sought to examine how condition, exploratory trial number, and age influence making diagnostic choices on the exploratory trials. The model included subject-specific random intercepts and random slopes for condition, trial, and their interaction term. We used sum-to-zero contrasts for the categorical variable *condition*, which captures deviation from the grand mean. The uncontrollable condition was coded as 0, and the controllable condition was coded as 1.

Full model: diagnostic exploratory choices ~ condition * (age + age-squared) * trial number + (1 + (condition * trial number | SubjID)

Table E. Diagnostic choice during exploratory trials

| Predictor | Regression Coefficient (*SE*) | *χ^2^* | *p* |
| --- | --- | --- | --- |
| **Intercept** | **.58 (.2)** |  |  |
| **Condition** | **.13 (.03)** | **16.05** | **< .0001** |
| Age | .28 (.15) | 3.6 | .06 |
| Age^2^ | -.27 (.15) | 3.09 | .08 |
| **Trial number** | **.17 (.06)** | **7.97** | **.005** |
| Condition x Age | .03 (.02) | 1.36 | .24 |
| Condition x Age^2^ | -.04 (.02) | 2.49 | .11 |
| Condition x Trial number | .05 (.02) | 3.7 | .05 |
| **Age x Trial number** | **.1 (.04)** | **5.24** | **.02** |
| Age^2^ x Trial number | .05 (.04) | 1.26 | .26 |
| Trial number x Age x Condition | .03 (.02) | 2.09 | .15 |
| Trial number x Age^2^ x Condition | -.01 (.02) | .08 | .78 |

**Working memory as a mediator between age and state prediction accuracy**

To better assess the specific role of working memory in predicting state transitions, we asked whether age-related differences in working memory might account for developmental improvements in state prediction accuracy. A mediation analysis revealed that working memory partially mediated the relationship between age and state prediction accuracy (standardized indirect effect: .08, 95% confidence interval: [.01 : .19], *p* = .011; standardized direct effect: .45, 95% confidence interval: [.27 : .61], *p* < .001 ), suggesting that working memory plays a role in making accurate predictions about the state that will be encountered next.
