# Supplementary material for: Developmental shifts in computations used to detect environmental controllability
Source: PLoS Comput Biol. 2022 Jun 1;18(6):e1010120. doi: 10.1371/journal.pcbi.1010120 (PMC9191713; doi:10.1371/journal.pcbi.1010120)
Supplement: S2 Appendix — Table A. Model comparison testing age vs. age2 for model-derived estimates (DOCX) [file pcbi.1010120.s006.docx]

Developmental shifts in computations

used to detect environmental controllability

Hillary A. Raab, Careen Foord, Romain Ligneul, & Catherine A. Hartley

**S2 Appendix.** Computational modeling specifications and analyses.

**Computational Modeling**

The Spectator model learns the probability of transitioning to each of the three states from every state ($P(s'|s)$). Thus, the Spectator model tracks transition probabilities for nine state-state transitions, including transitions back to the current island. The Spectator model is unable to represent controllable contingencies as it does not take into account actions. However, by only learning about state-state transitions, it learns uncontrollable contingencies quickly. After every exploratory trial and each state prediction trial with feedback, the transition probability linking the initial state to the subsequent state is updated (Eq. 1), as are the transitions that did not occur (Eq. 2). The extent to which learned probabilities are updated by the most recent transition is governed by the learning rate ($\alpha_{ss'}$).

Realized transitions:

$P(s'|s) \leftarrow P(s'|s) + {\alpha_{ss'}}(1-P(s'|s))$ (1)

Unrealized transitions:

$P(s'|s) \leftarrow P(s'|s) (1- {\alpha_{ss'}})$ (2)

This model has two free parameters: a learning rate and an inverse temperature parameter that controls how deterministic choices are with respect to the most likely transition.

The Actor model learns the probability of reaching the three states following any state-action pair ($P(s'|s,a)$). The Actor model tracks the transition probabilities of all 27 state-action pairs. Just like the Spectator model, this model updates the realized (Eq. 3) and unrealized transitions (Eq. 4). It also has two free parameters: a learning rate ($\alpha_{sas'}$) and an inverse temperature.

Realized transitions:

$P(s'|s,a) \leftarrow P(s'|s,a) + {\alpha_{sas'}}(1-P(s'|s,a))$ (3)

Unrealized transitions:

$P(s'|s,a) \leftarrow P(s'|s,a) (1- {\alpha_{sas'}})$ (4)

The remaining two models, the Learned Transition Structure model and the Task Set model, both dynamically estimate the causal influence of actions over state transitions by comparing predictions about subsequent transitions from the Spectator and Actor models. The Learned Transition Structure model updates the state-state and state-action-state transition probabilities from experience, whereas the Task Set model uses prior knowledge about the rules governing the task transition structure to infer the degree of controllability of the environment and make state predictions.

Model estimation was performed using a Variational Bayesian (VB) approach, as implemented in a well-validated toolbox [[1]](https://www.zotero.org/google-docs/?TSOatM). The maximum number of VB iterations was set to 32, the minimum relative increase in variational energy considered was set to 10^-5^ and the minimum absolute increase in free energy was set to 0.02. The starting values of hidden states were never updated.

The fitting procedure estimated a set of native parameters assumed to be drawn from Gaussian distributions, without any boundary constraints. These native parameters were subsequently transformed within the functions governing the evolution of hidden states and choices. We constrained all learning rate parameters to the [0,1] interval, with native prior means of 0 and native prior variance of 3 (hence approximating a flat distribution over the interval after sigmoid transformation). The inverse temperature parameter determining prediction consistency was untransformed and unconstrained, with a native prior mean of 0 and a native prior variance of 10.

When applicable, the slope and threshold parameters controlling controllability inference (i.e mapping from Ω to ω) were constrained to the [0, Inf] (native prior mean 0 and variance 10, before exponential transformation) and to the [-1,1] intervals (native prior mean 0 and variance 3 before stretched sigmoid transformation), respectively.

To ensure the interpretability of parameters and model selection, we conducted model and parameter recovery analyses. We generated data for simulated participants using the empirical distributions obtained for each free parameter. More specifically, we fit normal distributions to the full sample of observed best-fitting values for each parameter, and we subsequently drew values randomly from these normal distributions (grey bars and blue curves of S2a Fig illustrates the empirical and fit-normal distributions used for the Task Set model simulations). A dataset of 250 simulated participants was generated for each model. Next, we fit each of the four different models to each of the four datasets and performed Bayesian model comparison for each dataset. Model recoverability was estimated by computing a confusion matrix showing how frequently each generating model was selected as the best fitting model (S2b Fig).

For parameter recovery, we focused on the Task Set model, which was the most frequent model in the population, and computed the correlation coefficients linking the randomly-drawn generating parameters with the recovered parameters, on- and off-diagonal (S2c Fig). Since the performance of younger participants was worse, and their choices noisier, we replicated the analyses described above using only the empirical distributions from 8-12 year old participants (S2d-e Fig). For this complementary analysis, data from only 125 simulated participants were generated for each model.

**Model comparisons**

Model comparisons used the Bayesian Information Criterion estimated using the marginal likelihood obtained from the model fitting procedure. Model comparisons treated model attribution as a random-effect, assuming that different participants may rely on different models to solve the task. It was implemented using the Bayesian Model Comparison (BMC) routine implemented by the VBA toolbox, using flat priors. Note that the BMC approach operates on log-model evidence (BIC*_evidence_* = -0.5xBIC*_classical_*).

**Age differences in model-derived estimates**

We conducted linear regressions to examine how the parameter estimates that govern controllability inferences within the best-fitting Task Set model changed from childhood to adulthood. First, we tested whether age alone or the addition of an age-squared term provided a better fit for all model-derived estimates. For each model-derived estimate, we ran an ANOVA comparing the models. The degrees of freedom for these analyses are (1,87). For all model-derived estimates, a model including only a linear age term, rather than the addition of an age-squared term, provided the best fit (*p*’s > 0.3).

Table A. Model comparison testing age vs. age^2^ for model-derived estimates

| Model | *F* | *p* | *Age or Age^2^* |
| --- | --- | --- | --- |
| Learning rate | .99 | .32 | Age |
| Inverse temperature | .02 | .88 | Age |
| Bias | .10 | .76 | Age |
| Slope | .03 | .86 | Age |

We found that the learning rate ($\alpha_{\Omega}$) decreased with age (𝛽 = -0.03, s.e. = 0.01, *t*(88) = -2.22, *p* = 0.029, Cohen’s 𝑓^2^ = 0.06; S3b Fig), reflecting greater stability of controllability beliefs across development. Simulations of choice behavior revealed that lower learning rates, at the levels characteristic of adolescents and adults, were robust to the noise associated with stochastic transitions in the task and led to more accurate controllability inferences. The inverse temperature increased across development (𝛽 = 0.96, s.e. = 0.15, *t*(88) = 6.61, *p* < 0.001, Cohen’s 𝑓^2^ = 0.5; S3c Fig), indicating that older individuals made choices more consistent with transition predictions from the Task Set model. Developmental differences were not evident in either the bias or the slope of the sigmoidal function that transforms Ω to ⍵ (bias: 𝛽 = 0.01, s.e. = 0.03, *t*(88) = 0.37, *p* = 0.714, Cohen’s 𝑓^2^ = 0.002; slope: 𝛽 = 0.59, s.e. = 3.2, *t*(88) = 0.19, *p* = 0.853, Cohen’s 𝑓^2^ < 0.001; S3d-e Fig).

**Working memory as a mediator between age and the improvement in model fit between the Spectator model and more complex models**

Working memory mediated the relationship between age and improvement in fit (Δ BIC) from the Spectator to the Task Set model. We next tested whether this working memory mediation held true for the other models that also incorporated knowledge about actions and their consequences. A formal mediation analysis revealed that working memory partially mediated the relationship between age and relative advantage in fit for the Actor versus Spectator model (standardized indirect effect: .07, 95% confidence interval: [.002 : .18], *p* = .043; standardized direct effect: .32, 95% confidence interval: [.1 : .54], *p* = .005). In addition, working memory partially mediation age-related improvements in fit for the Learned Transition Structure compared to Spectator model (standardized indirect effect: .06, 95% confidence interval: [.003 : .14], *p* = .04; standardized direct effect: .32, 95% confidence interval: [.08 : .51], *p* = .008).

References

1. [Daunizeau, J., Adam, V., & Rigoux, L. (2014). VBA: A Probabilistic Treatment of Nonlinear Models for Neurobiological and Behavioural Data. *PLOS Computational Biology*, *10*(1), e1003441. https://doi.org/10.1371/journal.pcbi.1003441](https://www.zotero.org/google-docs/?PhFZFp)
